# In Vitro Assays for Diagnosis of Drug-Induced Nonsevere Exanthemas: A Systematic Review and Meta-Analysis

**DOI:** 10.1155/2022/2386654

**Published:** 2022-12-21

**Authors:** Szymon Drygala, Elzbieta Rdzanek, Grzegorz Porebski, Pawel Dubiela

**Affiliations:** ^1^Department of Regenerative Medicine, Medical University of Bialystok, Bialystok, Poland; ^2^Department of Experimental and Clinical Pharmacology, Centre for Preclinical Research and Technology (CePT), Medical University of Warsaw, Warsaw, Poland; ^3^Department of Clinical and Environmental Allergology, Jagiellonian University Medical College, Krakow, Poland; ^4^Department of Pathophysiology and Allergy Research, Medical University of Vienna, Vienna, Austria

## Abstract

Frequent mislabelled causal relationship between drug hypersensitivity reactions and culprit drugs reinforces the need for an accurate diagnosis. The systematic reviews and meta-analyses of *in vitro* assays published so far focused on immediate reactions and the most severe delayed reactions, while the most frequent drug-induced delayed reactions—nonsevere exanthemas—have been underestimated. We aim to fill this gap. A systematic review of studies on *in vitro* assays used in the diagnosis of nonsevere drug-induced delayed reactions was conducted following the methodology of Preferred Reporting Items for a Systematic Review and Meta-analysis of Diagnostic Test Accuracy Studies Statement. The EMBASE and PubMed databases were searched. We have included 33 studies from which we extracted the data, then performed meta-analysis where possible, or synthesised the evidence narratively. The quality of the analysed studies was assessed with the QUADAS-2 tool. The tests identified the most frequently were lymphocyte transformation test (LTT), ELISpot, and ELISA. In the meta-analysis carried out for LTT in reactions induce by beta-lactams, the pool estimate of sensitivity and specificity amounted to 49.1% (95% CI: 14.0%, 85.0%) and 94.6% (95% CI: 81.7%, 98.6%), respectively. The studies showed heterogeneity in study design and laboratory settings, which resulted in a wide range of specificity and sensitivity of testing.

## 1. Introduction

Drug hypersensitivity reactions (DHRs) are common and are important culprits for unsuccessful treatment or the necessity of applying second-choice pharmacotherapy in daily clinical practice. They represent not only a health problem but also a significant financial burden for affected individuals and health systems [1–3]. DHRs are clinically classified as immediate reactions (IRs) or nonimmediate/delayed reactions (NIRs) [3, 4]. IRs trigger a spectrum of symptoms from mild to severe, including urticaria, angioedema, or anaphylaxis [3]. NIRs include all the range of clinical manifestations from maculopapular exanthema (MPE) or fixed drug eruption (FDE) to severe cutaneous adverse reactions (SCARs) such as the Stevens-Johnson syndrome/toxic epidermal necrolysis (SJS/TEN), acute generalized exanthematous pustulosis (AGEP), or drug reaction with eosinophilia and systemic symptoms (DRESS) [5, 6].

As for other diseases, misdiagnosis is the major factor that increases the burden and costs of the disease for patients [7, 8]. Over recent years, mislabelling is showing to be a relevant problem in two opposite ways, including false labels of allergic and false labels of nonallergic. This reinforces the need for accurate diagnosis followed by appropriate management [3]. The sensitivity and specificity of individual tests or assays used in the diagnosis of drug-induced reactions (immediate and delayed) are topics that are frequently undertaken and frequently discussed in the literature [3, 9–11]. However, studies in which the authors attempted to perform a meta-analysis (MA) based on studies identified through a systematic review of medical databases are rare and focused on immediate reactions [12].

Sousa-Pinto et al. have recently published an excellent systematic review with meta-analysis dedicated to the accuracy of penicillin allergy diagnostic tests [12]. However, the field of delayed DHR is far underrepresented in this respect. We had previously published a detailed analysis of *in vitro* assays as potential diagnostic tests in SCARs [10], but a more in-depth analysis of this issue related to MPE and other mild and moderate clinical manifestations of delayed DHR is still lacking. Taking into account the high prevalence of these reactions in daily practice, the reliable and well-established diagnostic tool would facilitate the management of these clinical conditions. Therefore, we have undertaken to systematize the available knowledge of *in vitro* assays used to diagnose delayed reactions other than SCARs and in case of availability of suitable tests to carry out their MA.

## 2. Methods

### 2.1. Design

A systematic review of primary studies of *in vitro* tests used in the diagnosis of delayed DHR (other than SCARS) was conducted according to the Preferred Reporting Items for a Systematic Review and Meta-analysis of Diagnostic Test Accuracy Studies (PRISMADTA) Statement [13]. Drug-induced reactions were classified as delayed on the basis of judgement of the authors in the particular analysed publication.

### 2.2. Criteria for Considering Studies

Publications meeting the following criteria were included in the review: (1) the publication concerned the diagnosis of nonimmediate drug hypersensitivity reactions other than SCARS (i.e., SJS/TEN, DRESS, and AGEP); (2) the test was conducted *in vitro*; (3) the study was conducted in a population of at least 5 patients; (4) the results are presented in a form that allows the estimate of sensitivity, specificity, or positive/negative predictive value (PPV/NPV) with value given directly or estimated on the basis of the number of patients with a positive and negative test result; and (5) publication in English or Polish, which is the native language of the authors. The following publication types were excluded: letter to the editor, conference abstracts, books, and documents. No restrictions on the publication date were applied.

### 2.3. Search Strategy

The databases EMBASE and PubMed (Medline) were searched in December 2020, without limitation on publication date. The search strategy was created in PubMed using keywords related to drug hypersensitivity, delayed symptoms of drug allergies, and *in vitro* tests. It was next adapted for EMBASE but kept as similar as possible. To ensure that no important study was omitted, the references of the included works were verified in terms of their compliance with the subject of the study and the inclusion/exclusion criteria. Furthermore, in August 2020, a systematic review of the latest secondary studies consisting of reviews and systematic reviews (i.e., published after January 1, 2018) was carried out to find primary studies that were not identified in the main review. A detailed search strategy, inclusion criteria, and a description of selection process are available in Supplementary Data.

### 2.4. Study Selection

Publications were selected in two stages. First, the titles and abstracts of the articles retrieved were analysed, and a list of studies that initially met the inclusion criteria was developed. In the second stage, full-text copies were obtained and checked to qualify the studies for inclusion in the analysis. The selection was carried out by two independent authors (E.R. and S.D.). In case of disagreement of opinions during the verification of full texts, the final position was developed by consensus with the participation of a third party (P.D.).

### 2.5. Data Extraction

The key data from selected studies were extracted by one of the authors (E.R.) and then verified by another (S.D.). The extraction was carried out approaching a previously prepared and standardized form that included the following: (i) study characteristics (country, setting, and sampling method); (ii) population characteristics (number of patients and age group); (iii) information on drug hypersensitivity (drugs that trigger allergic reactions as reported by participants and clinical manifestation); (iv) information on the applied *in vitro* tests (type of test and threshold for the definition of positive result); and (v) results. If the study involved more than one group of drugs, the extraction was also carried out by the drug group. The endpoints assessed in this review have been selected a priori and included the sensitivity, specificity, or PPV/NPV of the test.

### 2.6. Methods of Synthesis

For each of the tests, we extracted the frequency with which a positive result occurred. On the basis of this data and information about the results of reference tests or the patient's medical history (confirmed symptoms of drug allergy), we determined true positive (TP), true negative (TN), false positive (FP), or false negative (FN) values for each test. Where such an estimation was not possible, the values of parameters such as sensitivity, specificity, or PPV/NPV presented in the publication were used. If the study involved subgroups (i.e., acute or postrecovery phase) or data was split by drug or hypersensitivity reaction, the results were obtained both for the general population and for each subgroup (if available). Studies were grouped and analysed by test type.

### 2.7. Quantitative Synthesis

If multiple studies assessing the same issue are available, the appropriate procedure to obtain pooled results is an MA. While a typical MA usually concerns one endpoint, in case of diagnostic tests, their assessment most often takes into account two parameters—sensitivity and specificity. Therefore, these points should be analysed together. For MA, we used an interactive web-based tool, MetaDTA (Diagnostic Test Accuracy Meta-Analysis v2.0, 15th March 2021) [14, 15], which takes into account the correlation between sensitivity and specificity. Individual study estimates for trials included in the MA are presented as forest plots, and MA results are presented as a hierarchical summary receiver-operating characteristic (HSROC) curve with false positive rate (1-specificity) on the *x*-axis and sensitivity on the *y*-axis. Random-effect approach was used. Point estimates and 95% confidence intervals (CI) were estimated. MA was conducted only for tests for which at least 4 studies with full data (TP, FN, TN, and FP) were available. To be included in the MA, the studies had to concern the same group of drugs (particular drugs could differ), to have the same cut-off definition for a positive result and similar symptoms of delayed drug allergies present in the included patients.

### 2.8. Assessment of Risk of Bias

The quality of the included studies was independently evaluated by 2 authors (S.D. and P.D.) using the QUADAS-2 tool to assess the accuracy of diagnostic tests [16]. This tool allows one to evaluate the test in terms of its applicability (patient selection, test index, and reference standard) and to assess the risk of bias in 4 domains (3 domains shown above, plus flow and timing). Possible inconsistencies in the assessment were resolved through discussion and consensus with another author (E.R.).

## 3. Results

**Figure 1:**
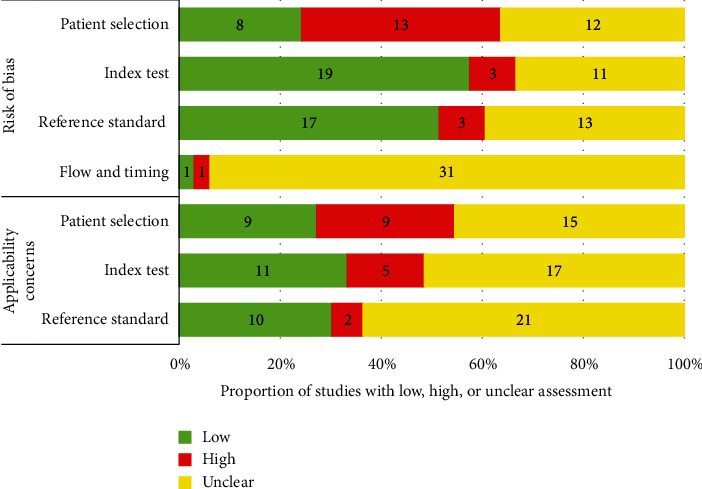
Quality of included primary studies assessed using QUADAS-2 tool.

A total of 650 records were identified from the search of medical databases, of which 571 were excluded on the basis of title and abstract. After removing duplicates, there were 79 records for which full texts were obtained, which were then assessed for eligibility. We found 16 primary studies that met the inclusion criteria with extractable data on *in vitro* tests used for the diagnosis of nonimmediate drug hypersensitivity reactions. Additional 17 studies were identified from the references and systematic review of secondary studies (for details, see Figure 1 Suppl.). In total, 33 primary studies were included in this analysis.

### 3.1. Study Characteristics

Characteristics of the included primary studies are presented in Table 1. Three trials [17–19] included children, and seven were conducted in children and adult population [20–24], while four lacked information on age in the patient's population [25–28]. The remaining studies included adults [29–39]. 23 studies were carried out in Europe [17, 23–27, 29–31, 33, 34, 39–47], and for one of the studies, information on where the trial was conducted was not available [48]. Most of the trials (28 out of 33) included a convenience sample of patients [17, 19–21, 23, 24, 26–28, 30–39, 41–49], while three had retrospective design [18, 25, 40], and two used the consecutive sample method [22, 29]. Information on the trial setting (inpatients and outpatients) was available only in two studies [20, 34].

### 3.2. Quality of Included Studies

The main issue with the quality of the primary studies included is the lack of sufficient information to determine whether the risk of bias is high or low. More than 30% of the studies were rated “unclear” in individual domains, and in case of flow and timing, this percentage reaches 94% of the studies. Additionally, the evaluation carried out on concerns related to the applicability of studies has a high percentage of studies rated “unclear”—depending on the domain, this percentage varies from 45% to 64%.  Figure 1 presents our assessment of the risk of bias and applicability concerns of the 33 primary studies included in the systematic review according to the QUADAS-2 tool.

### 3.3. Results of Individual Studies

The results of individual studies, divided by test type, are presented in Table 2.

### 3.4. Lymphocyte Proliferation/Transformation Tests (LTT)

The LTT results of 431 patients were extracted and pooled from the analysed studies (*n* = 24). The control comprised 318 LTT results from healthy individuals or LTT assays performed with irrelevant drugs in the patients. Three studies were conducted in the paediatric population, six studies were conducted in both the paediatric and adult populations, 13 studies described the results in the adult population, and four studies were carried out in an unknown population. Sensitivity in particular studies ranged from 0% to 100%, depending on the type of delayed hypersensitivity reaction and the drug analysed. The specificity ranged from 66.7% to 100%. The overall average of sensitivity and specificity of the LTT assay was, respectively, 48.6% and 93.7%. The sensitivity of the LTT assay for most of the drugs tested—beta-lactams and antiepileptics—ranged from 0% to 100% and 0% to 50%, respectively. The specificity range of LTT for those drug groups was as follows: 66.7% to 100% and 95.8% to 100%. The most frequently tested drugs were antibiotics (represented by beta-lactams) and antiepileptic drugs. Among the delayed hypersensitivity reactions, the most common was maculopapular exanthema (MPE). LTT seems to be the most accurate for detection of MPE caused by amoxicillin. Modifications in routine protocols with additional anti-CD3/anti-CD28 monoclonal antibody stimulation [47] and with B cells and monocytes or with dendritic cells serving as antigen presenting cells [43] revealed increased sensitivities, from 54.5% to 72.7% and from 22.2% to 88.9%, respectively. Data is collected in Table 2.

### 3.5. Enzyme-Linked Immunospot Assays (ELISpot)

ELISpot results from 415 patients were extracted and pooled from the analysed studies (*n* = 7), in which the assay was used to detect cells secreting IFN-*γ*, IL-4, IL-5, and GrB (Table 2). The control comprised 85 ELISpot results from healthy individuals or ELISpot assays performed with irrelevant drugs in patients. Most studies were carried out in the adult population (*n* = 4), and 1 study was carried out in the adult and paediatric population and 1 only in paediatric patients. In one of the studies, the age of the population has not been determined. The sensitivity in particular studies ranged from 0% to 100%, depending on the delayed hypersensitivity reaction type and the drug analysed. Specificity ranged from 82.9% to 100%; however, in only 4 studies, specificity was determined. The overall average of sensitivity and specificity of the LTT assay was 55.6% and 93.1%, respectively. The sensitivity of the ELISpot assay for most drugs tested, beta-lactams, represented by penicillins and antiepileptics represented by carbamazepine, lamotrigine, oxcarbazepine, and phenytoin ranged from 60% to 90.9% and 0% to 72.7%, respectively. The specificity range of ELISpot for those drug groups was the following: 82.9% to 95% and 95.8% to 100%. Among the delayed hypersensitivity reactions, the most common was MPE. The ELISpot test appears to be the most accurate for detection of MPE caused by penicillin (specificity: 90.9%; specificity: 95%). The value of this test for detection of penicillin-causing DHRs additionally confirms PPV and NPV with 100% and 84.6%, respectively.

In the papers by Polak et al. and Haw et al. [24, 40], ELISpot was used simultaneously for detection of various cytokines (IFN-*γ* and IL-4), which allows for direct comparison. ELISpot for IFN-*γ* and ELISpot for IL-4 showed very similar sensitivities in both studies (Table 2). In turn, the same cytokine detected with the ELISpot can provide significantly different results depending on the tested causal drugs, e.g., ELISpot for IFN-*γ* reached sensitivity 37.5% with antiepileptics [42] and 90.9% with beta-lactams [26].

### 3.6. Enzyme-Linked Immunosorbent Assay (ELISA)

ELISA experiments were performed with the measurement of the following cytokines: IL-5, IL-10, and IFN-*γ*. The experiments were designed to identify delayed allergic reactions only against antiepileptic drugs. The results of ELISA from 61 patients were extracted and pooled from the analysed studies (*n* = 4). The control comprised 61 ELISA results from healthy individuals or ELISA assays performed with irrelevant drugs in the patients. One study was carried out in mixed, paediatric and adult population, and the other 3 were carried out in adult population. Sensitivity in particular studies ranged from 17.4% to 91.7%, depending on a measured cytokine. The specificity ranged from 60% to 100%. The most specific biomarker was IL-5 with a sensitivity of 91.7% and a specificity of 100%. The overall average of sensitivity and specificity of the ELISA assay was 50.9% and 92%, respectively. Among the delayed hypersensitivity reactions, the only one under investigation was MPE (Table 2).

### 3.7. Basophil Activation Test (BAT)

BAT, as a well-known test for immediate allergic reactions, was also tested against delayed drug hypersensitivity in two studies. BAT assays were performed in three settings measuring the expression of CD203c+ and/or CD63+ to test allergic reactions against beta-lactams and antibiotics. The BAT results from 20 patients were extracted and pooled from the analysed studies (*n* = 2). The control consisted of 30 BAT results from healthy individuals. Sensitivity in particular studies ranged from 0% to 33.3%, depending on the expression measured activation marker, with CD63+ being more relevant for such a measurement. Specificity ranged from 78.6% to 100%. In a single study, CD63+ revealed 40% and 3.3% of positive and negative predictive values, respectively. In another study, where CD203c+ and CD63+ were applied, BAT had a negative predictive value of 53.3% (positive predictive value was impossible to calculate). Among the delayed hypersensitivity reactions, patients with MPE and benign skin rashes were tested with BAT (Table 2).

### 3.8. Other Tests

Other *in vitro* diagnostic assays identified in the literature were based on the detection of IFN-*γ* or intracellular staining followed by cytometric analysis of CD4+ cell proliferation [19]. Some other tests were based on the heparin-induced IgG assay [33] and radioallergosorbent test (RAST) known from the detection of immediate drug hypersensitivity reactions. Both the heparin-induced IgG assay and RAST do not show any suitability for DHR determination, which was confirmed by sensitivity results—22.2% and 0%, respectively. Cytometric analysis of CD4+ cell proliferation presented a high predictive value in the detection DHR. This was confirmed by the sensitivity and specificity results—100% and 90.9%, respectively. IFN-*γ* secretion appears to be an equally useful DHR determination test with a sensitivity of 71.4% and a 100% specificity [19]. Diagnostic parameters of these tests are shown in Table 2.

### 3.9. Meta-Analysis: LTT for Beta-Lactams

**Figure 2:**
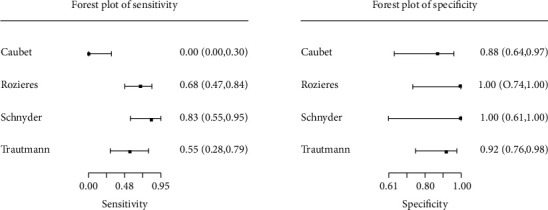
Meta-analysis of LTT (SI > 2.5). Forest plots of sensitivity and specificity.

**Figure 3:**
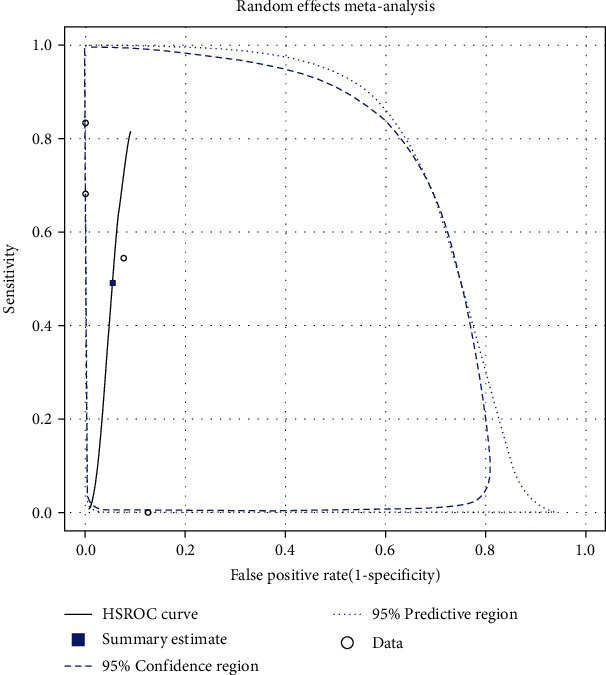
HSROC curve for the diagnostic performance of beta-lactam LTT in patients with delayed reactions.

An MA of studies reporting the detection of delayed allergies related to beta-lactams (the same cut-offs—stimulation index > 2.5) using LTT was conducted and comprised four studies [17, 26, 46, 47]. All these studies had been conducted in Europe and evaluated a reasonable sample of patients. One study [17] included children, two studies [45, 47] assessed adults, and one study [26] lacked information about the age of the participants. Three studies [17, 26, 46] investigated amoxicillin, and one study investigated other beta-lactams, cefuroxime [17], ticarcillin [26], and penicillin G [46], respectively. The fourth study tested ampicillin [47]. Studies included participants who reported benign skin rashes [17], MPE [26], exanthema [46], and macular or maculopapular exanthema [47]. Individual study estimates for each trial, both for sensitivity and specificity, are presented in Figure 2. Across these four studies, the pool estimate of sensitivity and specificity amounted to 49.1% (95% CI: 14.0%, 85.0%) and 94.6% (95% CI: 81.7%, 98.6%), respectively. The hierarchical summary receiver-operating characteristic curve for the diagnostic performance of beta-lactam LTT in patients with delayed reactions is shown in Figure 3.

## 4. Discussion

*In vitro* diagnostic tests in delayed DHR are considered difficult to perform and therefore limited to highly specialized centers. On the other hand, they would be highly useful to avoid time- and cost-consuming *in vivo* challenges (including drug provocation tests), facilitate allergologic diagnostic work-up in case of the patients living far from reference centers, and delabelling patients with a suspicion of drug allergy (positive lab test confirms allergy and changes a direction of a diagnostic pathway into alternative drugs). An important issue that needs to be emphasized considering *in vitro* tests is the fact that cytokines are determined in various conditions and various diseases [50]; however, only certain cytokines (i.e., TNF-*α*, IL-2, IL-4, IL-5, IL-6, IL-10, IL-13, and IFN-*γ*) are important in allergic reactions [51–56]. Delayed drug hypersensitivity reactions most often are mediated by IFN-*γ*, but in the case of reactions with a dominant role of eosinophils, IL-5 and IL-4/IL-13 play a major role. On the other hand, in reactions with a cytotoxic mechanism, such as SJS, perforin and granzyme B are of key importance. In turn, drug-induced reactions associated with neutrophilic inflammation, such as AGEP, seem to be associated with an increase in CXCL-8/GM-CSF [57]. We aimed to identify the most valuable and promising assay(s) for further development and application to daily practice. For practitioners working in the field of drug hypersensitivity, the burning questions regarding *in vitro* tests are how sensitive and specific those tests are in the detection of immune reaction to drugs implicated in DHR and how diagnostic parameters compare between *in vitro* tests?

In this systematic review, we evaluated the usefulness of *in vitro* tests for the diagnosis of delayed drug reactions other than SCARs. The exclusion of severe reactions such as SJS/TEN, AGEP, and DRESS was due to the fact that a more in-depth analysis of this issue addressing maculopapular exanthema and other mild clinical manifestations of delayed DHR is still lacking. Taking into account the high prevalence of these reactions in daily practice, a reliable and well-established diagnostic tool would facilitate the management of these clinical conditions. The systematic review covered a broad spectrum of tests and drugs; thus, despite a few differences between identified studies (included population, reported drug reactions, and associated risk of bias), the production of an MA of studies reporting the detection of delayed allergies related to beta-lactams (SI > 2.5) using LTT was possible. However, limitations of MA resulting from both low number of studies included, and the moderate heterogeneity should be taken into account while interpreting the pooled sensitivity and specificity of the test especially in a context of the patient population. Different response markers applied in parallel on the same platform may improve the overall test performance (i.e., both IFN-*γ* and IL-5 ELISpot [49]).

One of the major challenges in the diagnosis of DHR is the lack of standardized criteria for the evaluation of DHRs. Although there are a few testing possibilities, the criteria of the selection of the best diagnostic tools for each drug group are still missing. Among all tests utilized to diagnose DHR, the most studied group is LTT and its modification. The publications describe both paediatric [17, 18, 20–24, 29, 40] and adult groups [20–24, 29–49]. Also, broad drug spectrum was tested. The outcome of our analysis clearly highlights the lack of standardization in both the performance of the tests and the read out. It results in a wide range of specificity and sensitivity of testing. This, in turn, brings challenges to daily practice of physicians.

ELISpot is a commercially available method that applies an analysis of cytokine and other soluble molecule secretion from T-cell. The results of our analysis clearly demonstrate that there is also an immunological response, other than proliferation, available to use in the diagnosis of DHR. Seven studies included in our analysis have brought a range in specificity and sensitivity [24, 26, 31, 40–42, 49]. Although the specificity seemed to be much higher than for LTT, it is important to underline that only 4 studies included in the analysis aimed to measure it [24, 26, 41, 42]. There is also a limitation in the paediatric population that was represented only in 1 study [40]. Importantly, ELISpot was performed mostly in the detection of DHR in patients suffering from MPE [26, 40–42, 49].

ELISA [29, 32, 41, 42], BAT [17, 30], and other tests [19, 33, 44] had a very limited number of studies with the quality to be included in our systematic review. Again, as for the methods described above, the range in specificity and sensitivity suggests that the tests require further optimalization to be used as reliable for DHR detection. Of interest is the fact of high specificity in all three groups of tests (92% ELISA, 89.3% BAT, and 95.45% other tests). Unfortunately, the sensitivity of BAT [17, 30] and heparin-induced IgG assay [33] was below the results of LTT and ELISpot which underlines limitations of such methods.

Taking into consideration the ELISpot and ELISA tests, it has to be noted that the lack of standard cut-off values used in the discussed studies makes it difficult to directly compare their results. There are very different approaches in the literature to calculate thresholds in performance evaluation. For instance, a positive response can be calculated as a result above the upper limit of 95% confidence interval or higher than mean and 2 standard deviations calculated from samples serving as negative controls [11]. Therefore, well-designed methodological studies on the precise determination of threshold values for tests would provide crucial input for further development of these assays and their wider introduction into everyday practice.

It should be noted that despite the theoretical availability of many *in vitro* diagnostic methods, our meta-analysis indicates that the possibilities of using these methods in practice are limited. The most consistent evidence-based data relates first to LTT. The experience of a given diagnostic center in the field of cultivating PBMCs and stimulating them with suspected drugs is also of great importance, and this is a common step in different in vitro tests, regardless of the reading the systems used, such as LTT, ELISA, or ELISpot.

## 5. Conclusions

In summary, more specific and sensitive diagnostic tools are needed for a better patient management. Current testing brings uncertainty, and our systematic review does not provide a clear answer to the question which test should be used for each drug and patient group. LTT is most commonly used and has a good performance in beta-lactam-induced MPE. Based on that, it can be concluded that LTT seems to have the highest value in clinical practice among other *in vitro* tests. This is especially applicable for DHR detection in the adult population, as only single studies are available in small paediatric cohorts. Due to large heterogenicity in particular study results, the conclusion presented needs further investigation in well-designed studies conducted on large cohorts.

## Figures and Tables

**Table 1:** Characteristics of primary studies included in the systematic review.

Reference	Country	*n* participants	Participants' age group	Setting	Sampling method	Performed tests	Drug participants reported to be allergic
*Studies conducted in children population*							
Caubet et al. [17]	Switzerland	30	Children	Patients (not specified)	Convenience sample	LTT, BAT	Beta-lactams
Chopra et al. [18]	Canada	11	Children	Patients (not specified)	Retrospective	LTT	Amoxicillin
Tsuge et al. [19]	Japan	18	Children	Patients (not specified)	Convenience sample	CD4+ cell proliferation, IFN-*γ* secretion	Phenytoin
Haw et al. [40]	United Kingdom	16	Children and adults	Patients (not specified)	Retrospective	Lymphocyte proliferation assay (LPA), combination of cytokine assays	n/a

*Studies conducted in children and adult population*							
Ban et al. [20]	Korea	12	Children and adults	Inpatients and outpatients	Convenience sample	LTT	First-line antituberculosis medication (isoniazid, ethambutol, rifampin, and pyrazinamide)
Fu et al. [21]	China	6	Children and adults	Patients (not specified)	Convenience sample	LTT	Carbamazepine, acetaminophen, amoxicillin, penicillin, and cephazolin
Karami et al. [22]	Iran	48	Children and adults	Patients (not specified)	Consecutive sample	LTT	Anticonvulsant (phenobarbital, phenytoin, carbamazepine, and lamotrigine)
Orasch et al. [23]	Switzerland	20	Children and adults	Patients (not specified)	Convenience sample	LTT	Local anaesthetics
Polak et al. [24]	United Kingdom	57	Children and adults	Patients (not specified)	Convenience sample	ELISpot (IFN-*γ*, IL-4), LTT	Multiple drugs
Sachs et al. [29]	Germany	20	Children and adults	Patients (not specified)	Consecutive sample	ELISA (IL-5, IL-10, and IFN-*γ*), LTT	Multiple drugs

*Studies conducted in adult population*							
Abuaf et al. [30]	France	20	Adults	Patients (not specified)	Convenience sample	BAT	Amoxicillin
Ben-Said et al. [31]	France	56	Adults	Patients (not specified)	Convenience sample	IFN-*γ* ELISpot	Beta-lactams
Halevy and Grossman [32]	Israel	12	Adults	Patients (not specified)	Convenience sample	IFN-*γ* release test	Multiple drugs
Harenberg et al. [33]	Germany	9	Adults	Patients (not specified)	Convenience sample	Intracutaneous tests, HIPA assay, and heparin-induced IgG assay	LMW heparin
Hari et al. [34]	Switzerland	22	Adults	Inpatients and outpatients	Convenience sample	LTT	Multiple drugs
Kalish et al. [35]	USA	17	Adults	Patients (not specified)	Convenience sample	LTT	Trimethoprim-sulfamethoxazole, sulfapyridine, hydrochlorothiazide, and furosemide
Lopez et al. [36]	Spain	16	Adults	Patients (not specified)	Convenience sample	LTT	Heparins
Luque et al. [37]	Spain	47	Adults	Patients (not specified)	Convenience sample	RAST, LTT	Beta-lactams
Ónodi-Nagy et al. [38]	Hungary	10	Adults	Patients (not specified)	Convenience sample	LTT	Penicillins (amoxicillin, clavulanic acid, and cefixime)
Porebski and Czarnobilska [42]	Poland	47	Adults	Patients (not specified)	Convenience sample	ELISpot assay, cytometric analysis, LTT, ELISA	Antiepileptic drugs (carbamazepine, oxcarbazepine, lamotrigine, and phenytoin)
Porebski et al. [41]	Poland	32	Adults	Patients (not specified)	Convenience sample	Electrochemiluminescence assay (ECL), IFN-*γ*-ELISpot, LTT, IFN-*γ*-FACS, and IFN-*γ*-ELISA	Antiepileptic drugs (carbamazepine, oxcarbazepine, and lamotrigine)
Rodriguez-Pena et al. [43]	Spain	17	Adults	Patients (not specified)	Convenience sample	LTT	Amoxicillin
Romano [44]	Italy	24	Adults	Patients (not specified)	Convenience sample	RAST	Aminopenicillins
Schmid et al. [45]	Switzerland	6	Adults	Patients (not specified)	Convenience sample	LTT	Quinolones (ciprofloxacin, norfloxacin, moxifloxacin)
Schnyder and Pichler [46]	Switzerland	12	Adults	Patients (not specified)	Convenience sample	CAP, LTT	Amoxicillin and penicillin G
Srinoulprasert and Pichler [48]	Switzerland	6	Adults	Patients (not specified)	Convenience sample	Modified LTT, LTT	Amoxicillin, sulfamethoxazole, clavulanic acid, trimethoprim, and iomeron
Tanvarasethee et al. [49]	Bangkok	25	Adults	Patients (not specified)	Convenience sample	ELISpot	Ceftriaxone, ceftazidime
Trautmann et al. [47]	Germany	37	Adults	Patients (not specified)	Convenience sample	Modified LTT, LTT	Beta-lactams
Whitaker et al. [39]	United Kingdom	39	Adults	Patients (not specified)	Convenience sample	LTT	Piperacillin

*Studies conducted in unknown population*							
Nyfeler and Pichler [25]	Switzerland	102	n/a	Patients (not specified)	Retrospective	LTT	Multiple drugs
Rozieres et al. [26]	France	38	n/a	Patients (not specified)	Convenience sample	IFN-*γ* ELISpot, LTT	Amoxicillin, ticarcillin
Sachs et al. [27]	Germany	11	n/a	Patients (not specified)	Convenience sample	LTT	Aminopenicillins
Warrington and Tse [28]	Canada	12	n/a	Patients (not specified)	Convenience sample	LTT	Penicillin

BAT: basophil activation test; CAP: commercially available kit to penicilloyl G and penicilloyl V; ECL: electrochemiluminescence; ELISA: enzyme-linked immunosorbent assay; ELISpot: enzyme-linked immunospot assay; FACS: flow cytometric analysis; HIPA: heparin-induced platelet activation; IgG: immunoglobulin G; IFN-*γ*: interferon gamma; LPA: lymphocyte proliferation assay; LTT: lymphocyte transformation test; RAST: radioallergosorbent test.

**Table 2:** In vitro tests for the diagnosis of delayed drug allergies (other than SCARs).

Study	Test	Drugs tested	Hypersensitivity reactions	Subgroup	TP	TN	FP	FN	Sensitivity	Specificity	PPV	NPV	Test threshold for positive value
*LTT*													
Ban et al. [20]	LTT	First-line antituberculosis medication	MPE	Isoniazid	0			6	0.0%				SI ≥ 2
				Ethambutol	0			6	0.0%				
				Rifampin	3			3	50.0%				
				Pyrazinamide	0			6	0.0%				
				All drugs	3			21	12.5%				

Caubet et al. [17]	LTT	Beta-lactams (amoxicillin, cefuroxime)	Benign skin rashes	—	0	14	2	9	0.0%	87.5%	0.0%	60.9%	SI > 2.5

Chopra et al. [18]	LTT	Amoxicillin	Urticaria, angioedema, maculopapular rash, and erythema multiforme	—	2			9	18.2%				SI ≥ 2

Fu et al. [21]	LTT	Multiple drugs^1^	MPE	—	6			0	100.0%				n/a

Hari et al. [34]	LTT	Multiple drugs^2^	Bullous exanthema, MPE, and urticaria	—	14	15	1	7	66.7%	93.8%	93.3%	68.2%	SI > 2

Kalish et al. [35]	LTT	Multiple drugs^3^	Various types of rash	Sulfamethoxazole	4			12	25.0%				SI > 2
				Sulfisoxazole	3			8	27.3%				
				Furosemide	6			7	46.2%				
				Hydrochlorothiazide	1			3	25.0%				
				All drugs	14			30	31.8%				

Karami et al. [22]	LTT	anticonvulsant^4^	MPE	—	2	23	1	2	50.0%	95.8%	66.7%	92.0%	SI > 2

Lopez et al. [36]	LTT	Heparins	MPE, local reaction	All drugs	6	9	0	1	85.7%	100.0%	100.0%	90.0%	SI > 3

Luque et al. [37]	LTT	Beta-lactams (amoxicillin, penicillin G)	Exanthema, urticaria	All drugs	11	26	2	8	57.9%	92,9%	84,6%	76,5%	SI ≥ 3
				Benzyl-penicilloyl poly-L-lysine	6	27	1	13	31.6%	96.4%	85.7%	67.5%	
				Amoxicillin	10	27	1	9	52.6%	96.4%	90.9%	75.0%	

Nyfeler et al. [25]	LTT	Multiple drugs^5^	n/a	All drugs	78	87	15	22	78.0%	85.3%	83.9%	79.8%	SI > 2
				Penicillin	58	2	1	19	75.3%	66.7%	98.3%	9.5%	
				NSAIDs		68	9			88.3%			
				Beta-lactams					74.4%				

Ónodi-Nagy et al. [38]	LTT	Antibiotics/penicillins^6^	Maculopapular rash	All	1			9	10.0%				SI > 2
				Cefixime	1			0	100.0%				
				Amoxicillin/clavulanic acid	0			9	0.0%				

Orasch et al. [23]	LTT	Local anaesthetics	Angioedema, urticaria	—	6	6	0	4	60.0%	100.0%	100.0%	60.0%	SI > 2

Polak et al. [24]	LTT	Multiple drugs^7^	Drug-induced exanthems, eczema, and fixed drug eruption	DIE					33.3%				SI > 2
				Eczema					50.0%				
				FDE					100.0%				
				All reactions					36.6%	95.1%			

Porebski et al. [42]	LTT	Antiepileptic drugs^8^	MPE	All drugs	7	24	0	16	30.4%	100.0%	100.0%	60.0%	SI > 2
				Carbamazepine	4			8	33.3%				
				Oxcarbazepine	0			2	0.0%				
				Lamotrigine	3			5	37.5%				
				Phenytoin	0			1	0.0%				

Porebski et al. [41]	LTT	Antiepileptic drugs^9^	MPE	—	6			10	37.5%				SI > 2

Rodriguez-Pena et al. [43]	LTT	Amoxicillin	MPE	LTT	2	8	0	7	22.2%	100.0%	100.0%	53.3%	SI > 3
				LTT+DC	8	8	0	1	88.9%	100.0%	100.0%	88.9%	

Rozieres et al. [26]	LTT	Beta-lactams^10^	MPE	—	15	11	0	7	68.2%	100.0%	100.0%	61.1%	SI > 2.5

Sachs et al. [29]	LTT	Multiple drugs^11^	Macular-erythrodermic exanthem, MPE	All drugs	9			3	75.0%	100.0%			SI > 2.5
				Aminopenicillins	5			1	83.3%				
				Various drugs	4			2	66.7%				

Sachs et al. [27]	LTT	Aminopenicillins	Cytopenia, maculopapular exanthem, and skin eruption	—	10			1	90.9%				SI > 2.5

Schmid et al. [45]	LTT	Quinolones^12^	Exanthema, MPE	—	6			0	100.0%				n/a

Schnyder and Pichler [46]	LTT	Amoxicillin, penicillin G	Exanthema	—	10	6	0	2	83.3%	100.0%	100.0%	75.0%	SI ≥ 2.5

Srinoulprasert and Pichler [48]	LTT	Multiple drugs^13^	Anaphylaxis, late urticaria, and MPE	—	3			2	60.0%				SI > 2

Trautmann et al. [47]	LTT	Beta-lactams (ampicillin)	Macular or maculopapular exanthem	LTT	6	24	2	5	54.5%	92.3%	75.0%	82.8%	SI ≥ 2.5
				Modified LTT	8	20	6	3	72.7%	76.9%	57.1%	87.0%	

Warrington and Tse [28]	LTT	Penicillin	Maculopapular rashes	—	3			9	25.0%				n/a

Haw et al. [40]	Lymphocyte proliferation assay	Groups of drugs^∗^	MPE	Acute phase	5			1	83.3%				SI > 2

Whitaker et al. [39]	LTT	Piperacillin, tazocin	Mainly: MPE, fevers, urticarial eruptions, and flu-like symptoms	Postrecovery phase	1			2	33.3%				SI > 2
				MPE	10			5	66.7%				
				Piperacillin	4			5	44.4%				
				Tazocin	14			5	73.7%				

*ELISpot*													
Ben-Said et al. [31]	ELISpot (IFN-*γ*)	Beta-lactams	Exanthema	All drugs	6			6	50.0%				n/a
				Amoxicillin	6			4	60.0%				
				Cefazolin	0			1	0.0%				
				Imipenem	0			1	0.0%				

Haw et al. [40]	ELISpot (IFN-*γ*, IL-4)	Groups of drugs^∗^	MPE	IFN-*γ*, acute phase	5			1	83.3%				Responses greater than the mean of all the background samples plus 2 SD of the background
				IL-4, acute phase	4			0	100.0%				
				IFN-*γ*, postrecovery phase	3			0	100.0%				
				IL-4, postrecovery phase	3			0	100.0%				

Polak et al. [24]	ELISpot (IFN-*γ*, IL-4)	Multiple drugs^7^	Drug-induced exanthems, eczema, and fixed drug eruption	DIE, IFN-*γ*					51.9%				Responses greater than the mean of all the background samples plus 2 SD of the background
				Eczema, IFN-*γ*					100.0%				
				FDE, IFN-*γ*					100.0%				
				All IFN-*γ*					56.5%	82.9%			
				DIE, IL-4					59.1%				
				Eczema, IL-4					0.0%				
				FDE, IL-4					100.0%				
				All IL-4					56.6%	92.0%			

Porebski and Czarnobilska [42]	ELISpot (GrB)	Antiepileptic drugs^8^	MPE	All drugs	12	23	1	11	52.2%	95.8%	92.3%	67.6%	Δ values greater than the mean Δ value plus 2 SD measured in control subjects
				Carbamazepine	8			3	72.7%				
				Oxcarbazepine	1			1	50.0%				
				Lamotrigine	3			5	37.5%				
				Phenytoin	0			1	0.0%				

Porebski et al. [41]	ELISpot (IFN-*γ*)	Antiepileptic drugs^9^	MPE	—	6	16	0	10	37.5%	100.0%	100.0%	61.5%	A mean result plus 2 SD

Rozieres et al. [26]	ELISpot (IFN-*γ*)	penicillin^10^	MPE	IFN-*γ* ELISpot	20	11	0	2	90.9%	95.0%	100.0%	84.6%	Result above 30 IFN-c SFC/106 PBMC (mean plus 2 SD)

Tanvarasethee et al. [49]	ELISpot (IFN-*γ*, IL-5)	Ceftazidime, ceftriaxone	MPE	Both IFN-*γ* and IL-5	10			15	40.0%				The values greater than the means plus 2 SD of IFN-*γ* and IL-5 SFC/106 PBMCs
				IFN-*γ*	6			19	24.0%				
				IL-5	6			19	24.0%				

*ELISA*													
Porebski and Czarnobilska [42]	ELISA	Antiepileptic drugs^8^	MPE	All drugs	4	24	0	19	17.4%	100.0%	100.0%	55.8%	Δ values greater than the mean Δ value plus 2 SD measured in control subjects
				Carbamazepine	3			9	25.0%				
				Oxcarbazepine	0			2	0.0%				
				Lamotrigine	1			7	12.5%				
				Phenytoin	0			1	0.0%				

Porebski et al. [41]	ELISA (IFN-*γ*)	Antiepileptic drugs^9^	MPE	—	9	16	0	7	56.3%	100.0%	100.0%	69.6%	A mean result + 2 SD

Halevy and Grossman [32]	IFN-*γ* release	Multiple drugs	CADR	High drug suspicion	23			3	88.5%				
				Possible suspicion	2			0	100.0%				
				Low suspicion	7			7	50.0%				
				All drugs	32			10	76.2%				

Sachs et al. [29]	ELISA (IL-5, IL-10, IFN-*γ*)	Multiple drugs^11^	Macular-erythrodermic exanthem, MPE	18 challenge test (gold standard)					80.0%	62.0%	44.0%	89.0%	n/a
				All drugs	32			10	76.2%				

*BAT*													
Abuaf et al. [30]	BAT	Amoxicillin	Erythema, exfoliative dermatitis, and maculopapular rash	CD203c^+^	0	14	0	6	0.0%	100.0%	n/a	70.0%	2 SD (6%) exceeding the value of the nonstimulated control tube
				CD63^+^	2	11	3	4	33.3%	78.6%	40.0%	73.3%	
				CD203c^+^ or CD63^+^	2	11	3	4	33.3%	78.6%	40.0%	73.3%	

Caubet et al. [17]	BAT	Beta-lactams	Benign skin rashes	—	0	16	0	14	0.0%	100.0%	—	53.3%	SI (% of BL stimulation divided by % of negative control) >2 and the cut-off values for BL >7%

*Other*													
Harenberg et al. [33]	Heparin-induced IgG assay	Low-molecular-weight heparin	Red plaques, skin necrosis	—	2			7	22.2%				n/a

Romano [44]	RAST	Aminopenicillins	Maculopapular eruption	—	0			24	0.0%				n/a

Tsuge et al. [19]	CD4+ cell proliferation, IFN-*γ* secretion	Phenytoin	MPE	CD4+ cell proliferation	7	10	1	0	100.0%	90.9%	87.5%	100.0%	n/a
				IFN-*γ* secretion	5	11	0	2	71.4%	100.0%	100.0%	84.6%	

^1^Multiple drugs: acetaminophen, amoxicillin, carbamazepine, cephazolin, and penicillin; ^2^multiple drugs: acetazolamide, allopurinol, amoxicillin, carbamazepine, cefazolin, ceftriaxone, dorzolamide, indapamide, losartan, metolazone, penicillin G, prednisolone, simvastatin, sulfamethoxazole, ticlopidine, torasemide, trimethoprim, verapamil, and vit. B-complex; ^3^multiple drugs: furosemide, hydrochlorothiazide, sulfamethoxazole, and sulfisoxazole; ^4^anticonvulsant: phenobarbital and lamotrigine; ^5^multiple drugs: carbamazepine, ciprofloxacin, cotrimoxazole, diphenylhydantoin, erythromycin, lysozyme, methylephedrine-HCl, minocycline, penicillin, sulfadoxine-pyrimethamine, sulfamethoxazole, theophylline, and trimethoprim; ^6^antibiotics/penicillins: amoxicillin, clavulanic acid, and cefixime; ^7^multiple drugs: amoxicillin, aztreonam, bisoprolol fumarate, candesartan, carbamazepine, ceftazidime, ceftriaxone, cefuroxime, ciprofloxacin, clarithromycin, clindamycin, co-amoxiclav, codeine, doxycycline, erythromycin, flucloxacillin, fluconazole, gentamicin, hydroxychloroquine, itraconazole, levetiracetam, mercury, meropenem, omeprazole, oseltamivir, paracetamol, penicillin G, penicillin V, phenobarbital, phenytoin, Premarin, ramipril, senna, sodium valproate, tazocin, teicoplanin, tolterodine tartrate, trimethoprim, and vancomycin; ^8^antiepileptic drugs: carbamazepine, oxcarbazepine, lamotrigine, and phenytoin; ^9^antiepileptic drugs: carbamazepine, lamotrigine, and oxcarbazepine; ^10^beta-lactams: amoxicillin and ticarcillin; ^11^multiple drugs: amoxicillin, ampicillin, carbamazepine, clorazepate, fenoterol, phenobarbital, and phenytoin; ^12^quinolones: ciprofloxacin, moxifloxacin, and norfloxacin; ^13^multiple drugs: amoxicillin, sulfamethoxazole, clavulanic acid, trimethoprim, and iomeron; ^∗^groups of drugs: antibiotics, anticonvulsants, and antifungals. BAT: basophil activation test; CADR: cutaneous adverse drug reactions. CAP: commercially available kit to penicilloyl G and penicilloyl V; DIE: drug-induced exanthems; ECL: electrochemiluminescence; ELISA: enzyme-linked immunosorbent assay; ELISpot: enzyme-linked immunospot assay; FACS: flow cytometric analysis; FDE: fixed drug eruption; FN: false negative; FP: false positive; LTT: lymphocyte transformation test; MPE: maculopapular exanthema; PBMC: peripheral blood mononuclear cells; RAST: radioallergosorbent test; SD: standard deviation; SI: stimulation index; TN: true negative; TP: true positive.

## Data Availability

The data supporting this systematic review and meta-analysis are from previously reported studies and datasets, which have been cited. The processed data are available from the corresponding author on reasonable request to researchers who provide a methodologically sound proposal.

## Supplementary Materials

Supplementary Materials Supplementary Material.docx (Figure S1 and S2 and Table S1-S10).

## References

[1] Hsu D. Y., Brieva J., Silverberg N. B., Silverberg J. I. (2016). Morbidity and mortality of Stevens-Johnson syndrome and toxic epidermal necrolysis in United States adults. *The Journal of Investigative Dermatology*.

[2] Velasco-Tirado V., Alonso-Sardón M., Cosano-Quero A. (2018). Life-threatening dermatoses: Stevens-Johnson Syndrome and toxic epidermal necrolysis. Impact on the Spanish public health system (2010-2015). *PloS One*.

[3] Demoly P., Adkinson N. F., Brockow K. (2014). International consensus on drug allergy. *Allergy*.

[4] Thong B. Y., Tan T. C. (2011). Epidemiology and risk factors for drug allergy. *British Journal of Clinical Pharmacology*.

[5] Ojeda P., Sastre J., Olaguibel J. M., Chivato T., investigators participating in the National Survey of the Spanish Society of Allergology and Clinical Immunology Alergológica 2015 (2018). Alergólogica 2015: a national survey on allergic diseases in the adult Spanish population. *Journal of Investigational Allergology & Clinical Immunology*.

[6] Brockow K., Ardern-Jones M. R., Mockenhaupt M. (2019). EAACI position paper on how to classify cutaneous manifestations of drug hypersensitivity. *Allergy*.

[7] Jaoui A., Delalande D., Siouti S. (2019). Safety and cost effectiveness of supervised ambulatory drug provocation tests in children with mild non-immediate reactions to beta-lactams. *Allergy*.

[8] Mattingly T. J., Fulton A., Lumish R. A. (2018). The cost of self-reported penicillin allergy: a systematic review. *The Journal of Allergy and Clinical Immunology. In Practice*.

[9] Mayorga C., Celik G., Rouzaire P. (2016). In vitro tests for drug hypersensitivity reactions: an ENDA/EAACI Drug Allergy Interest Group position paper. *Allergy*.

[10] Porebski G. (2017). In vitro assays in severe cutaneous adverse drug reactions: are they still research tools or diagnostic tests already?. *International Journal of Molecular Sciences*.

[11] Porebski G., Piotrowicz-Wojcik K., Spiewak R. (2021). ELISpot assay as a diagnostic tool in drug hypersensitivity reactions. *Journal of Immunological Methods*.

[12] Sousa-Pinto B., Tarrio I., Blumenthal K. G. (2021). Accuracy of penicillin allergy diagnostic tests: a systematic review and meta- analysis. *The Journal of Allergy and Clinical Immunology*.

[13] McInnes M. D. F., Moher D., Thombs B. D. (2018). Preferred reporting items for a systematic review and meta-analysis of diagnostic test accuracy studies. *JAMA*.

[14] Freeman S. C., Kerby C. R., Patel A., Cooper N. J., Quinn T., Sutton A. J. (2019). Development of an interactive web-based tool to conduct and interrogate meta-analysis of diagnostic test accuracy studies: MetaDTA. *BMC Medical Research Methodology*.

[15] Patel A., Cooper N., Freeman S., Sutton A. (2021). Graphical enhancements to summary receiver operating characteristic plots to facilitate the analysis and reporting of meta-analysis of diagnostic test accuracy data. *Research Synthesis Methods*.

[16] Whiting Penny F., Rutjes Anne W. S., Westwood Marie E. (2011). QUADAS-2: a revised tool for the quality assessment of diagnostic accuracy studies. *Annals of Internal Medicine*.

[17] Caubet J.-C., Frossard C., Fellay B., Eigenmann P. A. (2015). Skin tests and in vitro allergy tests have a poor diagnostic value for benign skin rashes due to *β*-lactams in children. *Pediatric Allergy and Immunology*.

[18] Chopra R., Roberts J., Warrington R. J. (1989). Severe delayed-onset hypersensitivity reactions to amoxicillin in children. *CMAJ*.

[19] Tsuge I., Okumura A., Kondo Y. (2007). Allergen-specific T-cell response in patients with phenytoin hypersensitivity; simultaneous analysis of proliferation and cytokine production by carboxyfluorescein succinimidyl ester (CFSE) dilution assay. *Allergology International*.

[20] Ban G.-Y., Jeong Y.-J., Lee S.-H. (2019). Efficacy and tolerability of desensitization in the treatment of delayed drug hypersensitivities to anti-tuberculosis medications. *Respiratory Medicine*.

[21] Fu M., Gao Y., Pan Y. (2012). Recovered patients with Stevens-Johson syndrome and toxic epidermal necrolysis maintain long-lived IFN-*γ* and sFasL memory response. *PLoS One*.

[22] Karami Z., Mesdaghi M., Karimzadeh P. (2016). Evaluation of lymphocyte transformation test results in patients with delayed hypersensitivity reactions following the use of anticonvulsant drugs. *International Archives of Allergy and Immunology*.

[23] Orasch C. E., Helbling A., Zanni M. P., Yawalkar N., Hari Y., Pichler W. J. (1999). T-cell reaction to local anaesthetics: relationship to angioedema and urticaria after subcutaneous application-patch testing and LTT in patients with adverse reaction to local anaesthetics. *Clinical and Experimental Allergy*.

[24] Polak M. E., Belgi G., McGuire C. (2013). In vitro diagnostic assays are effective during the acute phase of delayed- type drug hypersensitivity reactions. *The British Journal of Dermatology*.

[25] Nyfeler B., Pichler W. J. (1997). The lymphocyte transformation test for the diagnosis of drug allergy: sensitivity and specificity. *Clinical and Experimental Allergy*.

[26] Rozieres A., Hennino A., Rodet K. (2009). Detection and quantification of drug-specific T cells in penicillin allergy. *Allergy*.

[27] Sachs B., Al Masaoudi T., Merk H. F., Erdmann S. (2004). Combined in vivo and in vitro approach for the characterization of penicillin- specific polyclonal lymphocyte reactivity: tolerance tests with safe penicillins instead of challenge with culprit drugs. *The British Journal of Dermatology*.

[28] Warrington R. J., Tse K. S. (1979). Lymphocyte transformation studies in drug hypersensitivity. *Canadian Medical Association Journal*.

[29] Sachs B., Erdmann S., Malte Baron J., Neis M., al Masaoudi T., Merk H. F. (2002). Determination of interleukin-5 secretion from drug-specific activated ex vivo peripheral blood mononuclear cells as a test system for the in vitro detection of drug sensitization. *Clinical and Experimental Allergy*.

[30] Abuaf N., Rostane H., Rajoely B. (2008). Comparison of two basophil activation markers CD63 and CD203c in the diagnosis of amoxicillin allergy. *Clinical and Experimental Allergy*.

[31] Ben-Said B., Arnaud-Butel S., Rozières A. (2015). Allergic delayed drug hypersensitivity is more frequently diagnosed in drug reaction, eosinophilia and systemic symptoms (DRESS) syndrome than in exanthema induced by beta-lactam antibiotics. *Journal of Dermatological Science*.

[32] Halevy S., Grossman N. (2008). Multiple drug allergy in patients with cutaneous adverse drug reactions diagnosed by in vitro drug-induced interferon-gamma release. *The Israel Medical Association Journal*.

[33] Harenberg J., Huhle G., Wang L., Hoffmann U., Bayerl C., Kerowgan M. (1999). Association of heparin-induced skin lesions, intracutaneous tests, and heparin-induced IgG. *Allergy*.

[34] Hari Y., Frutig-Schnyder K., Hurni M. (2001). T cell involvement in cutaneous drug eruptions. *Clinical and Experimental Allergy*.

[35] Kalish R. S., LaPorte A., Wood J. A., Johnson K. L. (1994). Sulfonamide-reactive lymphocytes detected at very low frequency in the peripheral blood of patients with drug-induced eruptions. *The Journal of Allergy and Clinical Immunology*.

[36] Lopez S., Torres M. J., Rodríguez-Pena R. (2009). Lymphocyte proliferation response in patients with delayed hypersensitivity reactions to heparins. *The British Journal of Dermatology*.

[37] Luque I., Leyva L., Torres M. J. (2001). *In vitro* T-cell responses to beta-lactam drugs in immediate and nonimmediate allergic reactions. *Allergy*.

[38] Ónodi-Nagy K., Kinyó Á., Meszes A., Garaczi E., Kemény L., Bata-Csörgő Z. (2015). Amoxicillin rash in patients with infectious mononucleosis: evidence of true drug *sensitization*. *Clinical Immunology*.

[39] Whitaker P., Meng X., Lavergne S. N. (2011). Mass spectrometric characterization of circulating and functional antigens derived from piperacillin in patients with cystic fibrosis. *Journal of Immunology*.

[40] Haw W. Y., Polak M. E., McGuire C., Erlewyn-Lajeunesse M., Ardern-Jones M. R. (2016). In vitro rapid diagnostic tests for severe drug hypersensitivity reactions in children. *Annals of Allergy, Asthma & Immunology*.

[41] Porebski G., Czarnobilska E., Bosak M. (2015). Cytotoxic-based assays in delayed drug hypersensitivity reactions induced by antiepileptic drugs. *Polskie Archiwum Medycyny Wewnętrznej*.

[42] Porębski G., Czarnobilska E. (2015). Drug-specific in vitro secretion of IFN*γ* in the diagnosis of drug-induced exanthemas: electrochemiluminescence assay versus previously used diagnostic methods. *Przegla̧d Lekarski*.

[43] Rodriguez-Pena R., Lopez S., Mayorga C. (2006). Potential involvement of dendritic cells in delayed-type hypersensitivity reactions to *β*-lactams. *The Journal of Allergy and Clinical Immunology*.

[44] Romano A. (1998). Delayed hypersensitivity to aminopenicillins. *Clinical and Experimental Allergy*.

[45] Schmid D. A., Depta J. P. H., Pichler W. J. (2006). T cell-mediated hypersensitivity to quinolones: mechanisms and cross- reactivity. *Clinical and Experimental Allergy*.

[46] Schnyder B., Pichler W. J. (2000). Skin and laboratory tests in amoxicillin- and penicillin-induced morbilliform skin eruption. *Clinical and Experimental Allergy*.

[47] Trautmann A., Seitz C. S., Stoevesandt J., Kerstan A. (2014). Aminopenicillin-associated exanthem: lymphocyte transformation testing revisited. *Clinical and Experimental Allergy*.

[48] Srinoulprasert Y., Pichler W. J. (2014). Enhancement of drug-specific lymphocyte proliferation using CD25^hi^ -depleted CD3^+^ effector cells. *International Archives of Allergy and Immunology*.

[49] Tanvarasethee B., Buranapraditkun S., Klaewsongkram J. (2013). The potential of using enzyme-linked immunospot to diagnose cephalosporin-induced maculopapular exanthems. *Acta Dermato-Venereologica*.

[50] Jurisic V. (2020). Multiomic analysis of cytokines in immuno-oncology. *Expert Review of Proteomics*.

[51] Gandhi V. D., Davidson C., Asaduzzaman M., Nahirney D., Vliagoftis H. (2013). House dust mite interactions with airway epithelium: role in allergic airway inflammation. *Current Allergy and Asthma Reports*.

[52] Nials A. T., Uddin S. (2008). Mouse models of allergic asthma: acute and chronic allergen challenge. *Disease Models & Mechanisms*.

[53] Vuolo F., Petronilho F., Sonai B. (2015). Evaluation of serum cytokines levels and the role of cannabidiol treatment in animal model of asthma. *Mediators of Inflammation*.

[54] Castan L., Magnan A., Bouchaud G. (2017). Chemokine receptors in allergic diseases. *Allergy*.

[55] Lambrecht B. N., Hammad H., Fahy J. V. (2019). The cytokines of asthma. *Immunity*.

[56] Silva R. A., Almeida F. M., Olivo C. R., Saraiva-Romanholo B. M., Martins M. A., Carvalho C. R. (2016). Exercise reverses OVA-induced inhibition of glucocorticoid receptor and increases anti-inflammatory cytokines in asthma. *Scandinavian Journal of Medicine & Science in Sports*.

[57] Pichler W. J. (2003). Delayed drug hypersensitivity reactions. *Annals of Internal Medicine*.

