# Supplementary material for: In Vitro Assays for Diagnosis of Drug-Induced Nonsevere Exanthemas: A Systematic Review and Meta-Analysis
Source: J Immunol Res. 2022 Dec 21;2022:2386654. doi: 10.1155/2022/2386654 (PMC9797304; doi:10.1155/2022/2386654)
Supplement: Supplementary Materials — Supplementary Material.docx (Figure S1 and S2 and Table S1-S10). [file 2386654.f1.docx]

# **Supplementary Material**

# **SYSTEMATIC REVIEW OF SECONDARY STUDIES**

A systematic review of the EMBASE and PubMed databases (last search was August 2020) was carried out to identify secondary studies of in vitro tests used in the diagnosis of non-immediate drug allergies (other than SCARS). The database search strategy is presented in the Supplementary Table. 4 and Supplementary Table. 5. The verification of abstracts and full texts of publications was carried out by 2 authors (E.R., S.D.). In case of disagreement of opinions, the final position was developed by consensus with a third party (P.D.).

A total of 371 abstracts were checked for compliance with the inclusion and exclusion criteria from the analysis (details below), of which 24 publications were qualified for verification based on the full text. Of these studies, 16 met the predefined inclusion criteria (see Supplementary Figure. 2). The list of included and excluded studies is presented in the Supplementary Table. 6 and Supplementary Table. 7, respectively.

## **Inclusion & exclusion criteria**

Secondary studies meeting the following inclusion criteria were included in the systematic review:

- Secondary study on the diagnosis of non-immediate drug allergies (other than SCARS).
- The review describes/refers to the studies that are related to in vitro tests.
- The review assessed at least one of the following endpoints (or made it possible to determine one of them based on TP, FP, TN, FN data):
- sensitivity,
- specificity,
- positive and negative predictive value.
- Studies published in the last 3 years (i.e. from 2018).
- Publication in Polish or English.

**Verification of the references of identified secondary studies**

First, references of secondary studies identified in the systematic review were checked in terms of meeting the criteria for inclusion in the review of primary studies or for the review of secondary studies (excluding the publication date). Then, references of secondary studies published before 2018 were also checked in terms of meeting inclusion & exclusion criteria for main review of primary studies.

Initially, a total of 55 new titles / abstracts were identified from among the references of 16 secondary studies which full texts were then verified taking into account inclusion & exclusion criteria. A total of 9 primary studies (meeting the criteria for inclusion in the main review) and 10 reviews published before 2018 were found (see Supplementary Table. 8). The list of excluded publications with reasons are presented in the Supplementary Table. 10.

In the next step, the references of 10 secondary studies (published before 2018) were checked, which allowed to identify another 8 primary studies that met the criteria for inclusion in the main review (see Supplementary Table. 9).

Overall, 17 primary studies that met the inclusion criteria for the main review were found.

**Supplementary Figure 1**. PRISMA flow diagram – systematic review of primary studies.

Records identified through database searching:

- MEDLINE: n = 498
- EMBASE: n = 152

Records screened

n = 650

Full-text articles assessed for eligibility after duplicates removed

n = 79

Full-text articles identified from the references and systematic review of secondary studies

n = 17

Studies included in qualitative synthesis

n = 33

Studies included in meta-analysis

n = 4

Full-text articles excluded, with reasons

n = 63

- Publication available as a conference abstract only (n = 23)
- Wrong publication type (n = 17)
- Publication subject inconsistent with the inclusion criteria (n = 13)
- No results that can be used (n = 8)
- Full text of the publication not available (n = 2)
- …

Records excluded

n = 571

**Supplementary Figure 2**. PRISMA flow diagram – systematic review of secondary studies.

Records identified through database searching:

- MEDLINE: n = 131
- EMBASE: n = 240

Records screened

n = 371

Full-text articles assessed for eligibility

n = 24

Secondary studies included in the analysis

n = 16

Full-text articles excluded, with reasons

n = 8

- No references that would meet the criteria for inclusion in the review of primary studies (n = 6)
- Full text of the publication not available (n = 2)

Primary studies identified from the references and included in the main review

n = 17

**Supplementary Table 1**. Search strategy for primary studies. PubMed (MEDLINE) database. Last search carried out on December 29, 2020.

| **Search** | **Query** | **Items found** |
| --- | --- | --- |
| 1 | allergy | 506,658 |
| 2 | reaction | 1,809,506 |
| 3 | hypersensitivity | 401,226 |
| 4 | #1 OR #2 OR #3 | 2,243,106 |
| 5 | drug | 5,963,748 |
| 6 | Immune-mediated | 21,418 |
| 7 | antiepileptics | 169,688 |
| 8 | betalactams | 20,721 |
| 9 | antibiotics | 944,992 |
| 10 | #5 OR #6 OR #7 OR #8 OR #9 | 6,351,067 |
| 11 | #4 AND #10 | 559,021 |
| 12 | DHR | 889 |
| 13 | #11 OR #12 | 559,785 |
| 14 | morbilliform drug eruptions | 156 |
| 15 | exanthematous drug eruption | 835 |
| 16 | maculopapular drug eruption | 26,607 |
| 17 | maculopapular exanthema | 559 |
| 18 | MPE | 2,350 |
| 19 | benign exanthems | 192 |
| 20 | exanthem | 10,129 |
| 21 | #14 OR #15 OR #16 OR #17 OR #18 OR #19 OR #20 | 37,638 |
| 22 | #13 AND #21 | 21,022 |
| 23 | non immediate | 42,229 |
| 24 | delayed | 570,832 |
| 25 | T-cell-mediated | 14,108 |
| 26 | #23 OR #24 OR #25 | 622,030 |
| 27 | #22 AND #26 | 1,147 |
| 28 | assay | 9,254,628 |
| 29 | test | 2,826,631 |
| 30 | diagnosis | 10,498,663 |
| 31 | diagnostic assay | 3,742,124 |
| 32 | #28 OR #29 OR #30 OR #31 | 16,894,533 |
| 33 | In vitro | 1,708,383 |
| 34 | #32 AND #33 | 1,024,315 |
| 35 | laboratory diagnosis | 2,886,635 |
| 36 | Lymphocyte transformation test | 14,573 |
| 37 | LTT | 784 |
| 38 | Lymphocyte proliferation test | 8,601 |
| 39 | ELISPOT | 6,573 |
| 40 | FACS | 15,281 |
| 41 | ELISA | 287,464 |
| 42 | #35 OR #36 OR #37 OR #38 OR #39 OR #40 OR #41 | 3,111,864 |
| 43 | #34 OR #42 | 3,756,561 |
| 44 | #27 AND #43 | 498 |

**Supplementary Table 2**. Search strategy for primary studies. EMBASE database. Last search carried out on December 29, 2020.

| **Search** | **Query** | **Items found** |
| --- | --- | --- |
| 1 | ('allergy'/exp OR allergy) AND [embase]/lim | 413,940 |
| 2 | reaction AND [embase]/lim | 2,999,050 |
| 3 | hypersensitivity AND [embase]/lim | 131,204 |
| 4 | #1 OR #2 OR #3 | 3,323,630 |
| 5 | drug AND [embase]/lim | 9,462,701 |
| 6 | 'immune mediated' AND [embase]/lim | 30,288 |
| 7 | antiepileptics AND [embase]/lim | 3,805 |
| 8 | betalactams AND [embase]/lim | 2,935 |
| 9 | antibiotics AND [embase]/lim | 255,327 |
| 10 | #5 OR #6 OR #7 OR #8 OR #9 | 9,550,869 |
| 11 | #4 AND #10 | 2,074,903 |
| 12 | dhr AND [embase]/lim | 1,255 |
| 13 | #11 OR #12 | 2,075,902 |
| 14 | morbilliform AND drug AND eruptions AND [embase]/lim | 116 |
| 15 | exanthematous AND drug AND eruption AND [embase]/lim | 779 |
| 16 | maculopapular AND drug AND eruption AND [embase]/lim | 1,787 |
| 17 | maculopapular AND exanthema AND [embase]/lim | 652 |
| 18 | mpe AND [embase]/lim | 3,072 |
| 19 | benign AND exanthems AND [embase]/lim | 17 |
| 20 | exanthem AND [embase]/lim | 1,194 |
| 21 | #14 OR #15 OR #16 OR #17 OR #18 OR #19 OR #20 | 7,017 |
| 22 | #13 AND #21 | 3,135 |
| 23 | non AND immediate AND [embase]/lim | 30,780 |
| 24 | delayed AND [embase]/lim | 346,480 |
| 25 | 't cell mediated' AND [embase]/lim | 18,947 |
| 26 | #23 OR #24 OR #25 | 391,384 |
| 27 | #22 AND #26 | 476 |
| 28 | assay AND [embase]/lim | 1,372,015 |
| 29 | test AND [embase]/lim | 2,631,819 |
| 30 | diagnosis AND [embase]/lim | 4,692,739 |
| 31 | diagnostic AND assay AND [embase]/lim | 107,783 |
| 32 | #28 OR #29 OR #30 OR #31 | 7,558,560 |
| 33 | in AND vitro AND [embase]/lim | 1,960,156 |
| 34 | #32 AND #33 | 511,297 |
| 35 | laboratory AND diagnosis AND [embase]/lim | 503,670 |
| 36 | lymphocyte AND transformation AND test AND [embase]/lim | 5,578 |
| 37 | LTT AND [embase]/lim | 1,035 |
| 38 | lymphocyte AND proliferation AND test AND [embase]/lim | 8,649 |
| 39 | elispot AND [embase]/lim | 10,104 |
| 40 | facs AND [embase]/lim | 33,744 |
| 41 | elisa AND [embase]/lim | 286,596 |
| 42 | #35 OR #36 OR #37 OR #38 OR #39 OR #40 OR #41 | 824,807 |
| 43 | #34 OR #42 | 1,243,177 |
| 44 | #27 AND #43 | 152 |

**Supplementary Table 3.** Studies identified from systematic review of primary studies.

| **Study** | **Reference** |
| --- | --- |
| Ban 2019 | Ban G-Y, Jeong Y-J, Lee S-H, Shin S-S, Shin Y-S, Park H-S, Kim S-H, Ye Y-M. Efficacy and tolerability of desensitization in the treatment of delayed drug hypersensitivities to anti-tuberculosis medications. *Respir Med* 2019;147:44-50. |
| Ben-Said 2015 | Ben-Said B, Arnaud-Butel S, Rozières A, Rodet K, Bérard F, Nicolas JF, Nosbaum A. Allergic delayed drug hypersensitivity is more frequently diagnosed in drug reaction, eosinophilia and systemic symptoms (DRESS) syndrome than in exanthema induced by beta-lactam antibiotics. *J Dermatol Sci* 2015;80(1):71-74. |
| Chopra 1989 | Chopra R, Roberts J, Warrington RJ. Severe delayed-onset hypersensitivity reactions to amoxicillin in children. *CMAJ* 1989;140(8):921-923. |
| Halevy 2008 | Halevy S, Grossman N. Multiple drug allergy in patients with cutaneous adverse drug reactions diagnosed by in vitro drug-induced interferon-gamma release. *Isr Med Assoc J* 2008;10(12):865-868. |
| Harenberg 1999 | Harenberg J, Huhle G, Wang L, Hoffmann U, Bayerl C, Kerowgan M. Association of heparin-induced skin lesions, intracutaneous tests, and heparin-induced IgG. *Allergy* 1999;54(5):473-477. |
| Karami 2016 | Karami Z, Mesdaghi M, Karimzadeh P, Mansouri M, Taghdiri MM, Kayhanidoost Z, Jebelli B, Shekarriz Foumani R, Babaie D, Chavoshzadeh Z. Evaluation of Lymphocyte Transformation Test Results in Patients with Delayed Hypersensitivity Reactions following the Use of Anticonvulsant Drugs. *Int Arch Allergy Immunol* 2016;170(3):158-162. |
| Onodi-Nagy 2015 | Ónodi-Nagy K, Kinyó, Meszes A, Garaczi E, Kemény L, Bata-Csörgo Z. Amoxicillin rash in patients with infectious mononucleosis: Evidence of true drug sensitization. *Allergy Asthma Clin Immunol* 2015;11(1):1. |
| Porebski 2015a | Porębski G, Czarnobilska E, Bosak M. Cytotoxic‑based assays in delayed drug hypersensitivity reactions induced by antiepileptic drugs. *Pol Arch Med Wewn* 2015;125(11):823-834. |
| Porebski 2015b | Porebski G, Czarnobilska E. Drug-specific in vitro secretion of IFNγ in the diagnosis of drug-induced exanthemas: electrochemiluminescence assay versus previously used diagnostic methods. *Przegl Lek* 2015;72(12):721-724. |
| Romano 1998 | Romano A. Delayed hypersensitivity to aminopenicillins. *Clin Exp Allergy* 1998;28(Suppl 4):29-32. |
| Rozieres 2009 | Rozieres A, Hennino A, Rodet K, Gutowski M.-C, Gunera-Saad N, Berard F, Cozon G, Bienvenu J, Nicolas J-F. Detection and quantification of drug-specific T cells in penicillin allergy. *Allergy* 2009;64(4):534-542. |
| Sachs 2004 | Sachs B, Al Masaoudi T, Merk HF, Erdmann S. Combined in vivo and in vitro approach for the characterization of penicillin-specific polyclonal lymphocyte reactivity: tolerance tests with safe penicillins instead of challenge with culprit drugs. *Br J Dermatol* 2004;151(4):809-816. |
| Schmid 2006 | Schmid DA, Depta JPH, Pichler WJ. T cell-mediated hypersensitivity to quinolones: Mechanisms and cross-reactivity. *Clin* *Exp Allergy* 2006;36(1):59-69. |
| Schnyder 2000 | Schnyder B, Pichler WJ. Skin and laboratory tests in amoxicillin- and penicillin-induced morbilliform skin eruption. *Clin* *Exp Allergy* 2000;30(4):590-595. |
| Trautmann 2014 | Trautmann A, Seitz CS, Stoevesandt J, Kerstan A. Aminopenicillin-associated exanthem: Lymphocyte transformation testing revisited. *Clin Exp Allergy* 2014;44(12)1531-1538. |
| Tsuge 2007 | Tsuge I, Okumura A, Kondo Y, Itomi S, Kakami M, Kawamura M, Nakajima Y, Komatsubara R, Urisu A. Allergen-specific T-cell response in patients with phenytoin hypersensitivity; simultaneous analysis of proliferation and cytokine production by Carboxyfluorescein Succinimidyl Ester (CFSE) dilution assay. *Allergol Int* 2007;56(2):149-155. |

**Supplementary Table 4**. Search strategy for primary studies. PubMed (MEDLINE) database. Last search carried out on August 8, 2020.

| **Search** | **Query** | **Items found** |
| --- | --- | --- |
| 1 | allergy | 495,164 |
| 2 | reaction | 1,765,527 |
| 3 | hypersensitivity | 390,300 |
| 4 | #1 OR #2 OR #3 | 2,189,140 |
| 5 | drug | 5,758,879 |
| 6 | Immune-mediated | 20,477 |
| 7 | antiepileptics | 164,624 |
| 8 | betalactams | 20,525 |
| 9 | antibiotics | 916,183 |
| 10 | #5 OR #6 OR #7 OR #8 OR #9 | 6,140,589 |
| 11 | #4 AND #10 | 540,303 |
| 12 | DHR | 850 |
| 13 | #11 OR #12 | 541,041 |
| 14 | assay | 8,974,153 |
| 15 | test | 2,719,435 |
| 16 | diagnosis | 10,189,560 |
| 17 | diagnostic assay | 3,604,637 |
| 18 | #14 OR #15 OR #16 OR #17 | 16,419,983 |
| 19 | In vitro | 1,670,142 |
| 20 | #18 AND #19 | 995,115 |
| 21 | #13 AND #20 | 47,263 |
| 22 | #21 Filters: Review, Systematic Review, from 2018 – 2020 | 131 |

**Supplementary Table 5**. Search strategy for primary studies. EMBASE database. Last search carried out on August 8, 2020.

| **Search** | **Query** | **Items found** |
| --- | --- | --- |
| 1 | (‘allergy’/exp OR allergy) AND [embase]/lim | 339,952 |
| 2 | reaction AND [embase]/lim | 2,902,591 |
| 3 | hypersensitivity AND [embase]/lim | 127,967 |
| 4 | #1 OR #2 OR #3 | 3,199,527 |
| 5 | drug AND [embase]/lim | 9,172,452 |
| 6 | ‘immune mediated’ AND [embase]/lim | 28,292 |
| 7 | antiepileptics AND [embase]/lim | 3,621 |
| 8 | betalactams AND [embase]/lim | 2,787 |
| 9 | antibiotics AND [embase]/lim | 243,801 |
| 10 | #5 OR #6 OR #7 OR #8 OR #9 | 9,256,608 |
| 11 | #4 AND #10 | 1,998,313 |
| 12 | dhr AND [embase]/lim | 1,323 |
| 13 | #11 OR #12 | 1,999,391 |
| 14 | assay AND [embase]/lim | 1,291,723 |
| 15 | test AND [embase]/lim | 2,547,982 |
| 16 | diagnosis AND [embase]/lim | 4,540,853 |
| 17 | diagnostic AND assay AND [embase]/lim | 100,408 |
| 18 | #14 OR #15 OR #16 OR #17 | 7,286,539 |
| 19 | in AND vitro AND [embase]/lim | 1,913,390 |
| 20 | #18 AND #19 | 456,608 |
| 21 | #13 AND #20 | 51,094 |
| 22 | #21 AND (2018:py OR 2019:py OR 2020:py) AND ‘review’/it | 240 |

**Supplementary Table 6**. Studies included in the systematic review of secondary studies.

| **Study** | **References** |
| --- | --- |
| Blanca-Lopez 2018 | Blanca-Lopez N, Somoza-Alvarez ML, Bellon T, Amo G, Canto G, Blanca M. NSAIDs hypersensitivity: questions not resolved. *Curr Opin Allergy Clin Immunol* 2018;18(4):291-301. |
| Blanca-Lopez 2019a | Blanca-Lopez N, Soriano V, Garcia-Martin E, Canto G, Blanca M. NSAID-induced reactions: classification, prevalence, impact, and management strategies. *J Asthma Allergy* 2019;12:217-233. |
| Blanca-Lopez 2019b | Blanca-Lopez N, Jimenez-Rodriguez TW, Somoza ML, Gomez E, Al-Ahmad M, Perez-Sala D, Blanca M. Allergic reactions to penicillins and cephalosporins: diagnosis, assessment of cross-reactivity and management. *Expert Rev Clin Immunol* 2019;15(7):707-721. |
| Brockow 2019 | Brockow K, Pfützner W. Cutaneous drug hypersensitivity: developments and controversies. *Curr Opin Allergy Clin Immunol* 2019;19(4):308-318. |
| Dona 2020 | Doña I, Pérez-Sánchez N, Eguiluz-Gracia I, Muñoz-Cano R, Bartra J, Torres MJ, Cornejo-García JA. Progress in understanding hypersensitivity reactions to nonsteroidal anti-inflammatory drugs. *Allergy* 2020;75(3):561-575. |
| Fernandez 2018 | Fernandez J, Doña I. Diagnosing and managing patients with drug hypersensitivity. *Expert Rev Clin Immunol* 2018;14(1):29-41. |
| Graham 2018 | Graham F, Tsabouri S, Caubet J-C. Hypersensitivity reactions to beta-lactams in children. *Curr Opin Allergy Clin Immunol* 2018;18(4):284-90. |
| Gregory 2020 | Gregory RJ, Woehlck H, Lien CA. Sugammadex and Hypersensitivity-Related Reactions: a Review. *Curr Anesthesiol Rep* 2020;10(2):123-30. |
| Hemmings 2018 | Hemmings O, Kwok M, McKendry R, Santos AF. Basophil Activation Test: Old and New Applications in Allergy. *Curr Allergy Asthma Rep* 2018;18(12):77. |
| Mayorga 2019a | Mayorga C, Ebo DG, Lang DM, Pichler WJ, Sabato V, Park MA, Makowska J, Atanaskovic-Markovic M, Bonadonna P, Jares E. Controversies in drug allergy: In vitro testing. *J Allergy Clin Immunol* 2019;143(1):56-65. |
| Mayorga 2019b | Mayorga C, Fernandez TD, Montañez MI, Moreno E, Torres MJ. Recent developments and highlights in drug hypersensitivity. *Allergy* 2019;74(12):2368-81. |
| McGee 2019 | McGee EU, Samuel E, Boronea B, Dillard N, Milby MN, Lewis SJ. Quinolone Allergy. *Pharmacy (Basel)* 2019;7(3):97. |
| Rukasin 2019 | Rukasin CRF, Norton AE, Broyles AD. Pediatric Drug Hypersensitivity. *Curr Allergy Asthma Rep* 2019;19(2):11. |
| Saretta 2019 | Saretta F, Mori F, Cardinale F, Liotti L, Franceschini F, Crisafulli G, Caimmi S, Bottau P, Bernardini R, Caffarelli C. Pediatric drug hypersensitivity: which diagnostic tests? *Acta Biomed* 2019;90(3-S):94-107. |
| Trautmann 2018 | Trautmann A. Wurpts G. Penicillin allergy – recommendations for diagnostic work up and patient management. *Allergo Journal International* 2018;27(4):107-113. |
| Wohrl 2018 | Wöhrl S. NSAID hypersensitivity - recommendations for diagnostic work up and patient management. *Allergo J Int* 2018;27(4):114-121. |

**Supplementary Table 7.** Studies excluded from the systematic review of secondary studies.

| **Study** | **References** | **Reason for exclusion** |
| --- | --- | --- |
| Garvey 2019 | Garvey LH, Savic LC. Drug provocation testing: risk stratification is key. *Curr Opin Allergy Clin Immunol* 2019;19(4):266-271. | no references that would meet the inclusion criteria for the systematic review of primary studies |
| Meng 2018 | Meng X, Yerly D, Naisbitt DJ. Mechanisms leading to T-cell activation in drug hypersensitivity. *Curr Opin Allergy Clin Immunol* 2018;18(4):317-324. | no references that would meet the inclusion criteria for the systematic review of primary studies |
| Mori 2020 | Mori F, Saretta F, Bianchi A, Crisafulli G, Caimmi S, Liotti L, Bottau P, Franceschini F, Paglialunga C, Ricci G, Santoro A, Caffarelli C. Hypersensitivity Reactions to Monoclonal Antibodies in Children. *Medicina (Kaunas)* 2020;56(5):232. | no references that would meet the inclusion criteria for the systematic review of primary studies |
| Nabhan 2020 | Nabhan M, Pallardy M, Turbica I. Immunogenicity of Bioproducts: Cellular Models to Evaluate the Impact of Therapeutic Antibody Aggregates. *Front Immunol* 2020 5;11:725. | no references that would meet the inclusion criteria for the systematic review of primary studies |
| Phillips 2019 | Phillips EJ, Bigliardi P, Bircher AJ, Broyles A, Chang YS, Chung WH, Lehloenya R, Mockenhaupt M, Peter J, Pirmohamed M, Roujeau JC, Shear NH, Tanno LK, Trubiano J, Valluzzi R, Barbaud A. Controversies in drug allergy: Testing for delayed reactions. *J Allergy Clin Immunol* 2019;143(1):66-73. | no references that would meet the inclusion criteria for the systematic review of primary studies |
| Takazawa 2019 | Takazawa T, Sabato V, Ebo DG. In vitro diagnostic tests for perioperative hypersensitivity, a narrative review: potential, limitations, and perspectives. *Br J Anaesth* 2019;123(1):e117-e125. | no references that would meet the inclusion criteria for the systematic review of primary studies |

**Supplementary Table 8**. Primary and secondary studies identified from references of secondary studies included in the analysis.

| **Study** | **References** |
| --- | --- |
| **Primary studies** | |
| Abuaf 2008 | Abuaf N, Rostane H, Rajoely B, Gaouar H, Autegarden JE, Leynadier F, Girot R. Comparison of two basophil activation markers CD63 and CD203c in the diagnosis of amoxicillin allergy. *Clin Exp Allergy* 2008;38(6):921-8. |
| Caubet 2015 | Caubet J-C, FrossardC, Fellay B, Eigenmann PA. Skin tests and in vitro allergy tests have a poor diagnostic value for benign skin rashes due to β-lactams in children. *Pediatr Allergy Immunol* 2015;26(1):80-2. |
| Haw 2016 | Haw WY, Polak ME, McGuire C, Erlewyn-Lajeunesse M, Ardern-Jones MR. In vitro rapid diagnostic tests for severe drug hypersensitivity reactions in children. *Ann Allergy Asthma Immunol* 2016;117(1):61-6. |
| Luque 2001 | Luque I, Leyva L, Torres MJ, Rosal M, Mayorga C, Segura JM, Blanca M, Juárez C. In vitro T-cell responses to beta-lactam drugs in immediate and nonimmediate allergic reactions. *Allergy* 2001;56(7):611-8. |
| Nyfeler 1997 | Nyfeler B, Pichler WJ. The lymphocyte transformation test for the diagnosis of drug allergy: sensitivity and specificity. *Clin Exp Allergy* 1997;27(2):175-81. |
| Orasch 1999 | Orasch CE, Helbling A, Zanni MP, Yawalkar N, Hari Y, Pichler WJ. T-cell reaction to local anaesthetics: relationship to angioedema and urticaria after subcutaneous application--patch testing and LTT in patients with adverse reaction to local anaesthetics. *Clin Exp Allergy* 1999;29(11):1549-54. |
| Polak 2013 | Polak ME, Belgi G, McGuire C, Pickard C, Healy E, Friedmann PS, Ardern-Jones MR. In vitro diagnostic assays are effective during the acute phase of delayed-type drug hypersensitivity reactions. *Br J Dermatol* 2013;168(3):539-49. |
| Rodriguez-Pena 2006 | Rodriguez-Pena R, Lopez S, Mayorga C, Antunez C, Fernandez TD, Torres MJ, Blanca M. Potential involvement of dendritic cells in delayed-type hypersensitivity reactions to beta-lactams. *J Allergy Clin Immunol* 2006;118(4):949-56. |
| Sachs 2002 | Sachs B, Erdmann S, Malte Baron J, Neis M, al Masaoudi T, Merk HF. Determination of interleukin-5 secretion from drug-specific activated ex vivo peripheral blood mononuclear cells as a test system for the in vitro detection of drug sensitization. *Clin Exp Allergy* 2002;32(5):736-44. |
| **Secondary studies** | |
| Dona 2017a | Doña I, Moreno E, Pérez-Sánchez N, Andreu I, Fernandez de Rojas DH, Torres MJ. Update on Quinolone Allergy. *Curr Allergy Asthma Rep* 2017;17(8):56. |
| Dona 2017b | Doña I, Torres MJ, Montañez MI, Fernández TD. In Vitro Diagnostic Testing for Antibiotic Allergy. *Allergy Asthma Immunol Res* 2017;9(4):288-298. |
| Ebo 2011 | Ebo DG, Leysen J, Mayorga C, Rozieres A, Knol EF, Terreehorst I. The in vitro diagnosis of drug allergy: status and perspectives. *Allergy* 2011 Oct;66(10):1275-86. |
| Gomez 2012 | Gómez E, Torres MJ, Mayorga C, Blanca M. Immunologic Evaluation of Drug Allergy. *Allergy Asthma Immunol Res* 2012;4(5):251–263. |
| Mayorga 2016 | Mayorga C, Celik G, Rouzaire P, Whitaker P, Bonadonna P, Rodrigues-Cernadas J, Vultaggio A, Brockow K, Caubet JC, Makowska J, Nakonechna A, Romano A, Montañez MI, Laguna JJ, Zanoni G, Gueant JL, Oude Elberink H, Fernandez J, Viel S, Demoly P, Torres MJ, In vitro tests for Drug Allergy Task Force of EAACI Drug Interest Group. In vitro tests for drug hypersensitivity reactions: an ENDA/EAACI Drug Allergy Interest Group position paper. *Allergy* 2016;71(8):1103-34. |
| Mayorga 2017 | Mayorga C, Doña I, Perez-Inestrosa E, Fernández TD, Torres MJ. The Value of In Vitro Tests to Diminish Drug Challenges. *Int J Mol Sci* 2017;18(6):1222. |
| McGowan 2012 | McGowan EC, Saini S. Update on the performance and application of basophil activation tests. *Curr Allergy* *Asthma Rep* 2013;13(1):101-9. |
| Pichler 2004 | Pichler WJ, Tilch J. The lymphocyte transformation test in the diagnosis of drug hypersensitivity. *Allergy* 2004;59(8):809-20. |
| Porebski 2011 | Porebski G, Gschwend-Zawodniak A, Pichler WJ. In vitro diagnosis of T cell-mediated drug allergy. *Clin Exp Allergy* 2011;41(4):461-70. |
| Torres 2017 | Torres MJ, Romano A, Celik G, Demoly P, Khan DA, Macy E, Park M, Blumenthal K, Aberer W, Castells M, Barbaud A, Mayorga C, Bonadonna P. Approach to the diagnosis of drug hypersensitivity reactions: similarities and differences between Europe and North America. *Clin Transl Allergy* 2017;7:7. |

**Supplementary Table. 9.** Primary studies identified from references of secondary studies published before 2018.

| **Study** | **References** |
| --- | --- |
| Fu 2012 | Fu M, Gao Y, Pan Y, Li W, Liao W, Wang G, Li C, Li C, Gao T, Liu Y. Recovered patients with Stevens-Johson syndrome and toxic epidermal necrolysis maintain long-lived IFN-γ and sFasL memory response. *PLoS One* 2012;7(9):e45516. |
| Hari 2001 | Hari Y, Frutig-Schnyder K, Hurni M, Yawalkar N, Zanni MP, Schnyder B, Kappeler A, von Greyerz S, Braathen LR, Pichler WJ. T cell involvement in cutaneous drug eruptions. *Clin Exp Allergy* 2001;31(9):1398-408. |
| Kalish 1994 | Kalish RS, LaPorte A, Wood JA, Johnson KL. Sulfonamide-reactive lymphocytes detected at very low frequency in the peripheral blood of patients with drug-induced eruptions. *J Allergy Clin Immunol* 1994;94(3 Pt 1):465-72. |
| Lopez 2009 | Lopez S, Torres MJ, Rodríguez-Pena R, Blanca-Lopez N, Fernandez TD, Antunez C, Canto G, de Luque V, Mayorga C. Lymphocyte proliferation response in patients with delayed hypersensitivity reactions to heparins. *Br J Dermatol* 2009;160(2):259-65. |
| Srinoulprasert 2014 | Srinoulprasert Y, Pichler WJ. Enhancement of Drug-Specific Lymphocyte Proliferation Using CD25 hi -Depleted CD3 + Effector Cells. *Int Arch Allergy Immunol* 2014;163:198–205. |
| Tanvarasethee 2013 | Tanvarasethee B, Buranapraditkun S, Klaewsongkram J. The potential of using enzyme-linked immunospot to diagnose cephalosporin-induced maculopapular exanthems. *Acta Derm Venereol* 2013;93(1):66-9. |
| Warrington 1979 | Warrington RJ, Tse KS. Lymphocyte transformation studies in drug hypersensitivity. *Can Med Assoc J* 1979;120(9):1089-94. |
| Whitaker 2011 | Whitaker P, Meng X, Lavergne SN, El-Ghaiesh S, Monshi M, Earnshaw C, Peckham D, Gooi J, Conway S, Pirmohamed M, Jenkins RE, Naisbitt DJ, Park BK. Mass spectrometric characterization of circulating and functional antigens derived from piperacillin in patients with cystic fibrosis. *J Immunol* 2011;187(1):200-11. |

**Supplementary Table 10**. Publications identified from references of secondary studies that did not meet the criteria for inclusion.

| Study | References | Reason for exclusion |  |
| --- | --- | --- | --- |
| Aberer 2003 | Aberer W, Bircher A, Romano A, Blanca M, Campi P, Fernandez J, Brockow K, Pichler WJ, Demoly P, European Network for Drug Allergy (ENDA); EAACI interest group on drug hypersensitivity. Drug provocation testing in the diagnosis of drug hypersensitivity reactions: general considerations. *Allergy* 2003;58(9):854-63. | Wrong publication type | |
| Aranda 2011 | Aranda A, Mayorga C, Ariza A, Doña I, Rosado A, Blanca-Lopez N, Andreu I, Torres MJ. In vitro evaluation of IgE-mediated hypersensitivity reactions to quinolones. *Allergy* 2011;66(2):247-54. | Publication subject inconsistent with the inclusion criteria (the study concerns immediate reactions) | |
| Ariza 2016 | Ariza A, García-Martín E, Salas M, Montañez MI, Mayorga C, Blanca-Lopez N, Andreu I, Perkins J, Blanca M, Agúndez JAG, Torres MJ. Pyrazolones metabolites are relevant for identifying selective anaphylaxis to metamizole. *Scientific Reports* 2016;6:23845. | Publication subject inconsistent with the inclusion criteria (the study concerns immediate reactions) | |
| Bavbek 2009 | Ben Said B, Berard F, Bienvenu J, Nicolas J-F, Rozieres A. Usefulness of basophil activation tests for the diagnosis of IgE-mediated allergy to quinolones. *Allergy* 2010;65(4):535-6. | Publication subject inconsistent with the inclusion criteria (the study concerns immediate reactions) | |
| Beeler 2008 | Beeler A, Zaccaria L, Kawabata T, Gerber BO, Pichler WJ. CD69 upregulation on T cells as an in vitro marker for delayed-type drug hypersensitivity. *Allergy* 2008;63(2):181-8. | No results that can be used (no separate results for MPE patients) | |
| Ben Said 2010 | Ben Said B, Berard F, Bienvenu J, Nicolas J-F, Rozieres A. Usefulness of basophil activation tests for the diagnosis of IgE-mediated allergy to quinolones. *Allergy* 2010;65(4):535-6. | Publication subject inconsistent with the inclusion criteria (the study concerns immediate reactions) | |
| Blanca 2001 | Blanca M, Mayorga C, Torres MJ, Reche M, Moya MC, Rodriguez JL, Romano A, Juarez C. Clinical evaluation of Pharmacia CAP System RAST FEIA amoxicilloyl and benzylpenicilloyl in patients with penicillin allergy. *Allergy* 2001;56(9):862-70. | Publication subject inconsistent with the inclusion criteria (the study concerns immediate reactions) | |
| Cai 2018 | Cai F, Lucas M, Yun J. Dapsone-induced drug reaction with eosinophilia and systemic symptoms associated with HLA-B*13:01. *Intern Med J* 2018;48(3):363-364. | Wrong publication type | |
| Colombo 2008 | Colombo S, Rauch A, Rotger M, Fellay J, Martinez R, Fux C, Thurnheer C, Günthard HF, Goldstein DB, Furrer H, Telenti A, Swiss HIV Cohort Study. The HCP5 single-nucleotide polymorphism: a simple screening tool for prediction of hypersensitivity reaction to abacavir. *J Infect Dis* 2008;198(6):864-7. | Publication subject inconsistent with the inclusion criteria (the study does not concern in vitro test) | |
| de Paramo 2009 | de Paramo BJ, Gancedo SQ, Cuevas M, Camo IP, Martin JA, Cosmes EL. Paracetamol (acetaminophen) hypersensitivity. *Ann Allergy Asthma Immunol* 2000;85(6 Pt 1):508-11. | Wrong publication type | |
| De Weck 2009 | De Weck AL, Sanz ML, Gamboa PM, Aberer W, Blanca M, Correia S, Erdman S, Jermann JM, Kanny G, Kowalski M, Mayorga L, Medrala W, Merk A, Sturm GJ, Sainte-Laudy J, Schneider MS, Sczczeklik A, Weber JM, Wedi A. Nonsteroidal anti-inflammatory drug hypersensitivity syndrome. A multicenter study. I. Clinical findings and in vitro diagnosis. *J Investig Allergol Clin Immunol* 2009;19(5):355-69. | No results that can be used (lack of separate data for delayed reactions) | |
| Dewachter 2018 | Dewachter P, Chollet-Martin S, Mouton-Faivre C, de Chaisemartin L, Nicaise-Roland P. Comparison of Basophil Activation Test and Skin Testing Performances in NMBA Allergy. *J Allergy Clin Immunol Pract* 2018;6(5):1681-1689. | Publication subject inconsistent with the inclusion criteria (the study concerns perioperative allergy) | |
| Ebo 2007 | Ebo DG, Venemalm L, Bridts CH, Degerbeck F, Hagberg H, De Clerck LS, Stevens WJ. Immunoglobulin E antibodies to rocuronium: a new diagnostic tool. *Anesthesiology* 2007;107(2):253-9. | Publication subject inconsistent with the inclusion criteria (the study concerns immediate reactions) | |
| Fernandez 2016 | Fernández TD, Ariza A, Palomares F, Montañez MI, Salas M, Martín-Serrano A, Fernández R, Ruiz A, Blanca M, Mayorga C, Torres MJ. Hypersensitivity to fluoroquinolones: The expression of basophil activation markers depends on the clinical entity and the culprit fluoroquinolone. *Medicine (Baltimore)* 2016;95(23):e3679. | Publication subject inconsistent with the inclusion criteria (the study concerns immediate reactions) | |
| Gamboa 2003 | Gamboa PM, Sanz ML, Caballero MR, Antépara I, Urrutia I, Jáuregui I, González G, Diéguez I, De Weck AL. Use of CD63 expression as a marker of in vitro basophil activation and leukotriene determination in metamizol allergic patients. *Allergy* 2003;58(4):312-7. | Publication subject inconsistent with the inclusion criteria (the study concerns immediate reactions) | |
| Gamboa 2004 | Gamboa P, Sanz ML, Caballero MR, Urrutia I, Antépara I, Esparza R, de Weck AL. The flow-cytometric determination of basophil activation induced by aspirin and other non-steroidal anti-inflammatory drugs (NSAIDs) is useful for in vitro diagnosis of the NSAID hypersensitivity syndrome. *Clin Exp Allergy* 2004;34(9):1448-57 | Publication subject inconsistent with the inclusion criteria (the study concerns immediate reactions) | |
| Garvey 2007 | Garvey LH, Krøigaard M, Poulsen LK, Skov PS, Mosbech H, Venemalm L, Degerbeck F, Husum B. IgE-mediated allergy to chlorhexidine. *J Allergy Clin Immunol* 2007;120(2):409-15. | No results that can be used (no separate results for patients with delayed reactions) | |
| Hjortlund 2013 | Hjortlund J, Mortz CG, Skov PS, Bindslev-Jensen C. Diagnosis of penicillin allergy revisited: the value of case history, skin testing, specific IgE and prolonged challenge. *Allergy* 2013;68(8):1057-64. | No results that can be used (some patients had AGEP) | |
| Hoffmann 2015 | Hoffmann HJ, Santos AF, Mayorga C, Nopp A, Eberlein B, Ferrer M, Rouzaire P, Ebo DG, Sabato V, Sanz ML, Pecaric-Petkovic T, Patil SU, Hausmann OV, Shreffler WG, Korosec P, Knol EF. The clinical utility of basophil activation testing in diagnosis and monitoring of allergic disease. *Allergy* 2015;70(11):1393-405. | Wrong publication type | |
| Houwerzijl 1977 | Houwerzijl J, De Gast GC, Nater JP, Esselink MT, Nieweg HO. Lymphocyte-stimulation tests and patch tests to carbamazepine hypersensitivity. *Clin Exp Immunol* 1977;29(2):272-7. | Publication subject inconsistent with the inclusion criteria (no information on the type of reaction) | |
| Kano 2007 | Kano Y, Hirahara K, Mitsuyama Y, Takahashi R, Shiohara T. Utility of the lymphocyte transformation test in the diagnosis of drug sensitivity: dependence on its timing and the type of drug eruption. *Allergy* 2007;62(12):1439-44. | Publication subject inconsistent with the inclusion criteria (no information on the type of reaction) | |
| Kato 2017 | Kato K, Kawase A, Azukizawa H, Hanafusa T, Nakagawa Y, Murota H, Sakaguchi S, Asada H, Katayama I. Novel interferon-γ enzyme-linked immunoSpot assay using activated cells for identifying hypersensitivity-inducing drug culprits. *J Dermatol Sci* 2017;86(3):222-229. | No results that can be used (only one patient with MPE) | |
| Kowalski 2013 | Kowalski ML, Asero R, Bavbek S, Blanca M, Blanca-Lopez N, Bochenek G, Brockow K, Campo P, Celik G, Cernadas J, Cortellini G, Gomes E, Niżankowska-Mogilnicka E, Romano A, Szczeklik A, Testi S, Torres MJ, Wöhrl S, Makowska J. Classification and practical approach to the diagnosis and management of hypersensitivity to nonsteroidal anti-inflammatory drugs. *Allergy* 2013;68(10):1219-32. | Wrong publication type | |
| Kvedariene 2006 | Kvedariene V, Kamey S, Ryckwaert Y, Rongier M, Bousquet J, Demoly P, Arnoux B. Diagnosis of neuromuscular blocking agent hypersensitivity reactions using cytofluorimetric analysis of basophils. *Allergy* 2006;61(3):311-5. | Publication subject inconsistent with the inclusion criteria (the study concerns immediate reactions) | |
| Leysen 2011a | Leysen J, Bridts CH, De Clerck LS, Ebo DG. Rocuronium‐induced anaphylaxis is probably not mitigated by sugammadex: evidence from an in vitro experiment. *Anaesthesia* 2011;66(6):526-7. | Wrong publication type | |
| Leysen 2011b | Leysen J, Bridts CH, De Clerck LS, Vercauteren M, Lambert J, Weyler JJ, Stevens WJ, Ebo DG. Allergy to rocuronium: from clinical suspicion to correct diagnosis. *Allergy* 2011;66(8):1014-9. | Publication subject inconsistent with the inclusion criteria (the study concerns immediate reactions) | |
| Leysen 2014 | Leysen J, Uyttebroek A, Sabato V, Bridts CH, De Clerck LS, Ebo DG. Predictive value of allergy tests for neuromuscular blocking agents: tackling an unmet need. *Clin Exp Allergy* 2014;44(8):1069-75. | Publication subject inconsistent with the inclusion criteria (the study concerns perioperative allergy) | |
| Martin 2010 | Martin M, Wurpts G, Ott H, Baron JM, Erdmann S, Merk HF, Sachs B. In vitro detection and characterization of drug hypersensitivity using flow cytometry. *Allergy* 2010;65(1):32-9. | No results that can be used (some patients had SJS/TEN) | |
| Mockenhaupt 2009 | Mockenhaupt M. Severe drug-induced skin reactions: clinical pattern, diagnostics and therapy. *J Dtsch Dermatol Ges* 2009;7(2):142-60; quiz 161-2. | Wrong publication type | |
| Monneret 2002 | Monneret G, Benoit Y, Debard AL, Gutowski MC, Topenot I, Bienvenu J. Monitoring of basophil activation using CD63 and CCR3 in allergy to muscle relaxant drugs. *Clin Immunol* 2002;102(2):192-9. | Publication subject inconsistent with the inclusion criteria (the study concerns immediate reactions) | |
| Romano 2014 | Romano A, Caubet J-C. Antibiotic allergies in children and adults: from clinical symptoms to skin testing diagnosis. *J Allergy Clin Immunol Pract* 2014;2(1):3-12. | Wrong publication type | |
| Rouzaire 2012 | Rouzaire P, Nosbaum A, Denis L, Bienvenu F, Bérard F, Cozon G, Bienvenu J. Negativity of the basophil activation test in quinolone hypersensitivity: a breakthrough for provocation test decision-making. I*nt Arch Allergy Immunol* 2012;157(3):299-302. | Publication subject inconsistent with the inclusion criteria (the study concerns immediate reactions) | |
| Samuelov 2019 | Samuelov L, Nathan A, Slutsky E, Fruchter D, Gat A, Sprecher E, Goldberg I. Nested case-control study investigating the diagnostic role of tissue eosinophilia in adverse cutaneous drug reactions. *J Eur Acad Dermatol Venereol* 2019;33(6):1152-1157. | Publication subject inconsistent with the inclusion criteria (no information on the type of reaction) | |
| Seitz 2009 | Seitz CS, Bröcker EB, Trautmann A. Diagnostic testing in suspected fluoroquinolone hypersensitivity. *Clin Exp Allergy* 2009;39(11):1738-45. | Wrong publication type | |
| Torres 2009 | Torres MJ , Mayorga C, Blanca M. Nonimmediate allergic reactions induced by drugs: pathogenesis and diagnostic tests. *J Investig Allergol Clin Immunol* 2009;19(2):80-90. | Wrong publication type | |
| Zawodniak 2010 | Zawodniak A, Lochmatter P, Yerly D, Kawabata T, Lerch M, Yawalkar N, Pichler WJ. In vitro detection of cytotoxic T and NK cells in peripheral blood of patients with various drug-induced skin diseases. *Allergy* 2010;65(3):376-84. | No results that can be used (patients with a priori positive LTT were selected for the study) | |
